# Between Protein Fold and Nucleophile Identity: Multiscale Modeling of the TEV Protease Enzyme–Substrate Complex

**DOI:** 10.1021/acsomega.2c05201

**Published:** 2022-10-27

**Authors:** Alexander Zlobin, Andrey Golovin

**Affiliations:** † Belozersky Institute of Physico-Chemical Biology, Lomonosov Moscow State University, 119991 Moscow, Russia; ‡ Shemyakin and Ovchinnikov Institute of Bioorganic Chemistry, Russian Academy of Sciences, 117997 Moscow, Russia; § Sirius University of Science and Technology, 354340 Sochi, Russia

## Abstract

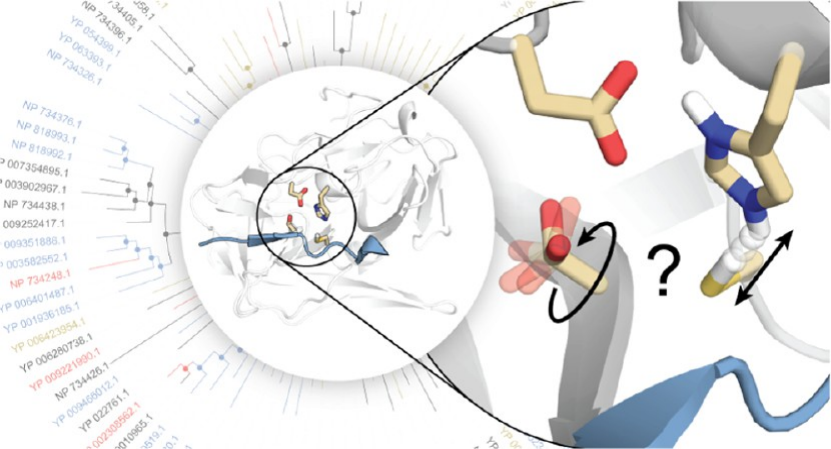

The cysteine protease from the tobacco etch virus (TEVp) is a well-known and widely utilized enzyme. TEVp’s chymotrypsin-like fold is generally associated with serine catalytic triads that differ in terms of a reaction mechanism from the most well-studied papain-like cysteine proteases. The question of what dominates the TEVp mechanism, nucleophile identity, or structural composition has never been previously addressed. Here, we use enhanced sampling multiscale modeling to uncover that TEVp combines the features of two worlds in such a way that potentially hampers its activity. We show that TEVp cysteine is strictly in the anionic form in a free enzyme similar to papain. Peptide binding shifts the equilibrium toward the nucleophile′s protonated form, characteristic of chymotrypsin-like proteases, although the cysteinyl anion form is still present and interconversion is rapid. This way cysteine protonation generates enzyme states that are a diversion from the most effective course of action, with only 13.2% of Michaelis complex sub-states able to initiate the reaction. As a result, we propose an updated view on the reaction mechanism catalyzed by TEVp. We also demonstrate that AlphaFold is able to construct protease–substrate complexes with high accuracy. We propose that our findings open a way for its industrious use in enzymological tasks. Unique features of TEVp discovered in this work open a discussion on the evolutionary history and trade-offs of optimizing serine triad-associated folds to cysteine as a nucleophile.

## Introduction

Proteases are enzymes capable of catalyzing the hydrolysis of peptide bonds.^1^ They do it differently by harboring a variety of catalytic site architectures in a variety of folds. The catalytic capabilities of proteases make them central to natural metabolism as well as biotechnology.

A cysteine protease from the tobacco etch virus (TEV protease, or simply TEVp) is one of the most frequently used proteases in the latter context.^2^ It is instrumental due to a number of appealing features. TEVp has a simple monomeric structure of just 27 kDa without any cofactors. Moreover, TEVp is highly sequence-specific with a natural predisposition to a seven amino acid sequence ENLYFQ|G/S. It should be noted, however, that such notation does not mean that G/S is a C-terminus, since TEVp is essentially an endopeptidase that cleaves itself out of the viral polyprotein. This opens the possibility of using TEVp as a controllable release-by-cleavage agent in biotechnological applications.^3^ It has been widely explored, leading to the emergence of complex tools with different modes of activation.^4−7^

With the biotechnological use of an enzyme comes a need to improve or adjust its properties. Variants with improved solubility and yield,^8,9^ reduced self-cleavage,^10^ and improved oxidative stability^11^ as well as immobilization strategies were reported.^12^ However, the biggest interest lies in the improvement of TEVp catalytic activity, since the wild type is slow compared to other widely used proteases that commonly implement serine as a nucleophile. To date, several directed evolution studies have yielded a handful of variants with up to an order of magnitude higher activity.^13,14^

No rational computational design has yet been applied to any TEVp property. Such approaches rely on the detailed knowledge of the exact events along the reaction pathway, that is, the mechanism.^15^ Rational computational design strategies were previously successfully used to yield enzymes with higher catalytic activity, including PETases,^16^ organophosphatases,^17^ cephalosporin acylases,^18^ and KEMP eliminases.^19^ Substrate specificity can also be engineered computationally.^20,21^ When applied to TEVp, it may yield a family of high-specific tag cleavage agents that pose interest to biotechnology. However, the main concurrent task is not to damage catalytic activity, since TEVp is already slow. Thus, this objective yet again requires detailed knowledge of the catalytic mechanism. Applying this knowledge to previously discovered variants will allow us to extract concrete origins of improved activity and build upon them.^22^ This knowledge also has a fundamental side. By learning why beneficial substitutions did not occur naturally, we can gain a better understanding of evolutionary factors specific to proteolytic enzymes in general.

**Figure 1:**
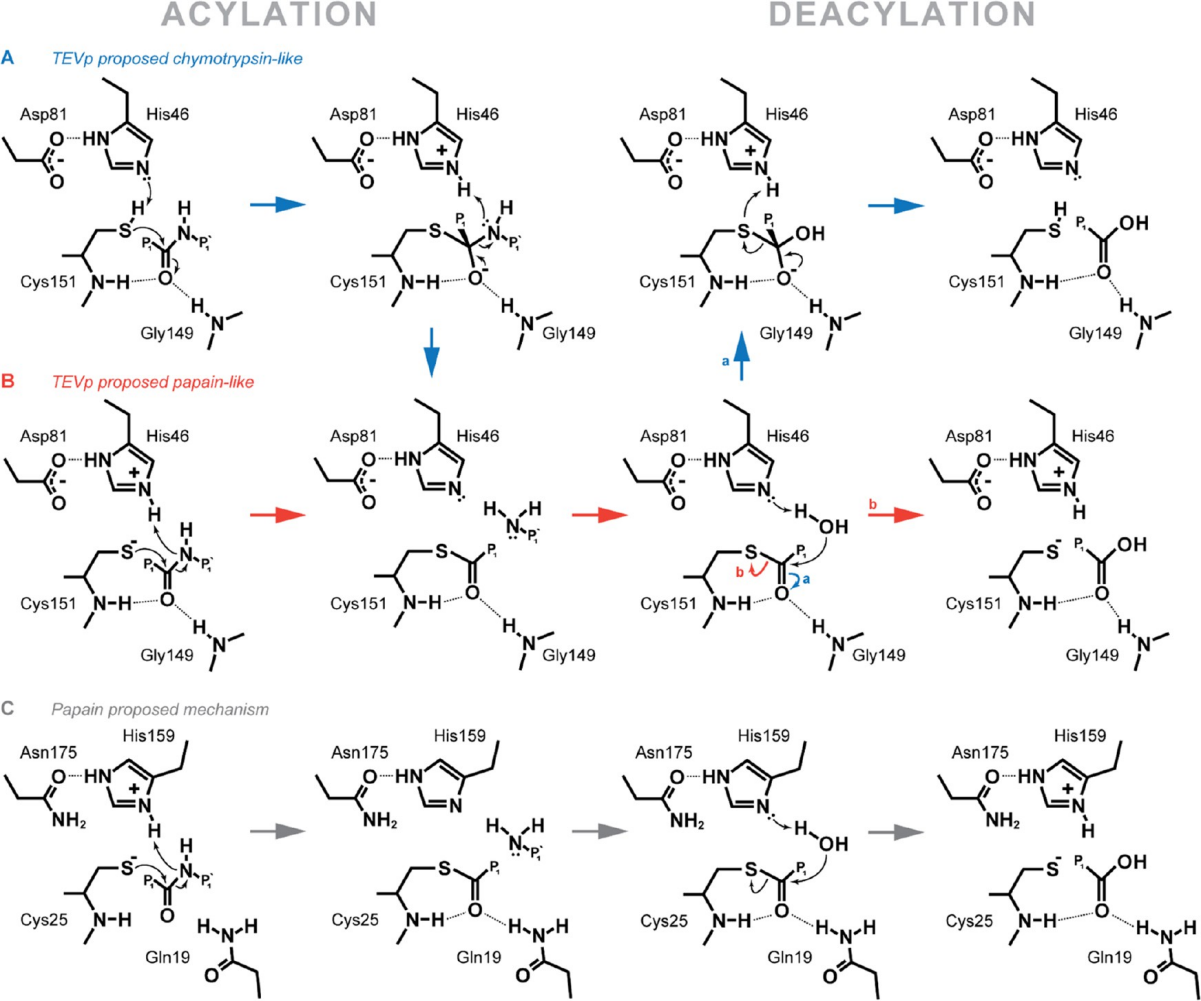
Variations of the proposed catalytic mechanisms of TEVp and a canonical papain mechanism. (A) Chymotrypsin-like mechanism as proposed for TEVp. Corresponding transitions are shown in blue. (B) Papain-like mechanism as proposed for TEVp. Corresponding transitions are shown in red. Capital letters indicate the starting states of corresponding processes. Lowercase letters indicate the bifurcation in the process most likely for each mechanism. (C) Generic papain mechanism proposed based on QM/MM studies.

Hypotheses about the TEVp mechanism were only ever postulated, not demonstrated in simulations. Major differences in assumptions concern the acylation stage and preceding events. Views diverge on what protonation state is characteristic of catalytic Cys and His at the Michaelis (enzyme–substrate) complex of TEVp and related proteases.^23^ It is not a cosmetic difference, since it results in a different mechanism overall (Figure 1). It is also intertwined with the nature of the products; however, this question is discussed much less often.

TEVp belongs to the PA clan of proteases and shares a structural similarity with chymotrypsin-like proteases that harbor the canonic Ser–His–Asp triad. This similarity reaches the very orientation of catalytic triad residues in space, despite TEVp having Cys instead of Ser as a nucleophile.^24^ As a result, TEVp is sometimes assumed to follow the same catalytic mechanism as Ser-utilizing PA clan proteases. These enzymes catalyze acylation, which is a rate-limiting stage, through a tetrahedral intermediate stabilized by interactions with oxyanion hole H-bond donors (Figure 1A). Ser is protonated at the Michaelis complex and deprotonation occurs alongside the formation of the tetrahedral intermediate.

The other proposed mechanism for TEVp, papain-like, follows the fact that the nucleophile is a cysteine (Figure 1B), not a serine. Papain-like proteases are the most well-studied cysteine proteases and are commonly believed to have a Cys in the deprotonated anionic state at the Michaelis complex.^23,25,26^ Activation barriers for these enzymes were found to be roughly equal for the acylation and deacylation stages. The acylation part of the papain-catalyzed reaction was most commonly shown to be absent of the tetrahedral intermediate, with proton transfer to peptide nitrogen occurring alongside S–C(pept) bond formation and C(pept)-N(pept) bond breakage (Figure 1C).^26−28^ Oxyanion hole in papain was shown not to interact with the substrate at the Michaelis complex but only in the transition state and afterward. However, TEVp is very different in terms of both the tertiary structure and catalytic site architecture from papain-like proteases (Figure S1).

The question that arises is as follows: what dominates the TEVp mechanism, nucleophile identity, or structural composition? Such a question may be answered with the help of molecular simulations.^29^ In this work, we perform a first-time-ever molecular simulation of the TEV protease. We focus our attention on the structure of the Michaelis complex with an optimal peptide substrate as a necessary starting point for further advancement. We conclude that there exists an equilibrium of Cys protonation states at the Michaelis complex of TEVp, while without a bound substrate, Cys is strictly deprotonated. Our attention was also drawn to the unusual conformation of a Ser residue that commonly interacts with catalytic Asp in PA clan proteases. In any other PA clan protease X-ray structure, this Ser faces outward with the associated N–CA–CB–OG torsion close to 180°. In TEVp X-ray structures, however, it faces inward, and the torsion value is close to −80°. We found that TEVp Ser may adopt a 180°-like conformation in dynamics and investigated the influence of such transition on the equilibrium of substrates within the Michaelis complex. With regard to this Ser, the TEVp protease turned out to differ even from the most closely related tobacco vein mottling virus (TVMV) protease. We discuss the structural origins of such a difference; however, to discover its functional meaning, modeling of the whole reaction process for both enzymes shall be performed in the future.

## Materials and Methods

### Model Building

The TEVp complex with the ENLYFQSGTV peptide was constructed with AlphaFold2-multimer-v2 (AFm)^30^ implemented in ColabFold^31^ v. 1.2.0. Multiple sequence alignment was built in ColabFold with MMSeqs2.^32^ Three recycles for all five models were used, and the best prediction according to the multimer score was picked for further work. Built-in energy minimization in Amber was not performed. For an expert assessment of model reliability, the 1LVM X-ray model of TEVp was taken.^33^ It also served as a donor of positions of coordinated water molecules.

PDB entries closest to TEVp in terms of fold were identified using the protein structure comparison service PDBeFold at European Bioinformatics Institute.^34,35^ The TVMVp complex with the NNVRFQSLDTIV peptide and the chymotrypsin C complex with the NIEVLEGNEQ peptide were built similarly, with reference X-ray models 3MMG and 4H4F, respectively.^36,37^ Cleavable peptide sequences were identified through MEROPS.^38^ AFm models used in the study are available in the Supporting Information. For kallikrein 4 (KLK4), an X-ray model of a complex with a modified sunflower trypsin inhibitor was taken (4KEL^39^) with Ala reverted to catalytic Ser using the mutagenesis tool in PyMol.^40^ For TEVp, TVMVp, and chymotrypsin C without a peptide, mentioned X-ray models were used as starting points. For KLK4, the 7JQO model was used.^41^ TEVp-T180V and TVMVp-V180T were generated using the mutagenesis tool in PyMol.

Four starting models for TEVp, both bound and free, were constructed: with either protonated/deprotonated Cys or X-ray/AFm Ser168 conformation to be subjected to molecular simulations and analysis together to prevent the initial state bias. For all other proteins, two starting models were constructed with different conformations of the Ser residue corresponding to Ser168 in TEVp (see the Results section).

For all models, PROPKA was used to generate hypotheses about residue protonation states in corresponding optimal pH ranges.^42^ Histidine tautomer assignment and correction of amide flips were verified manually on top of Molprobity recommendations.^43^

The SARS-Cov-2 main protease (MPro) complex with the ATVRLQAGNA peptide was built similarly to other systems. For reference, PDB model 7TA4 was taken.^44^ For runs with trimmed peptides, initial peptide sequences after P2′ were deleted, resulting in FQSGTV for TEVp and LQAGNA for SARS-Cov-2 MPro.

Trimmed alignments for AlphaFold were constructed as follows: The TEVp sequence was retained and supplemented with 20 random sequences from the initial alignment. Thirty such alignments were generated and submitted to AlphaFold.

### Simulation Software

All simulations were performed with Gromacs.^45^ For MM simulations, we used Gromacs 2021.3. For QM/MM simulations, we used an interface between Gromacs and DFTB+.^46,47^ Both were patched with Plumed 2.8.0.^48^

### MM Model Preparation and Equilibration

All previously listed models were parameterized in the Amber19 force field^49^ ported from Amber to Gromacs notation in-house.^50^ Each system was placed in a cubic box with periodic boundary conditions and solvated with tip3p-FB.^51^ All crystallographic water molecules were retained, while all added were manually filtered in case of incorrect solvation. Na^+^ and Cl^–^ ions were added to neutralize the net charge and reach 0.15 M ionic strength. Systems were minimized with 5000 steps of the steepest descent. The equilibration phase consisted of seven steps and was performed in three replicates for each combination “protease-Ser conformation-bound/free”, resulting in 12 runs per protease. First, an NVT run of 100 ps was performed while positionally restraining heavy atoms by 1000 kJ/mol/nm^2^. A velocity rescale thermostat was utilized^52^ for temperature coupling. Then, for five rounds of NPT equilibration of 100 ps each, restraint strength was gradually decreased as follows: 1000, 500, 200, 100, and 10 kJ/mol/nm^2^. A stochastic cell-rescale barostat was used for pressure control.^53^ A time step of 2 fs was used in all steps.

### QM/MM Model Preparation

QM/MM modeling was performed only for free and bound TEVp models. An intrabackbone boundary between QM and MM regions cannot be done in Amber19sb due to cmap usage to describe rotation in the phi/psi space instead of bond-specific torsion potentials. Thus, equilibration results were reparameterized in Amber99sb-ildn.^54^ Coordinates after the first NPT step were used as starting to prevent dealing with already manifested drift due to possibly unrealistic protonation. The QM subsystem consisted of 149 and 49 atoms (Figure S2) for bound and free systems, respectively. To prevent link atom hyperpolarization, a “charge shifting” scheme was used. The QM region was described with DFTB3^55^ with the 3ob-3-1 parameter set^56^ similarly to what was used before in various enzymological tasks.^57−61^ Additionally, D4 dispersion correction^62^ was utilized.

Systems were subjected to 1000 steps of conjugate gradient minimization and 10000 steps of unrestrained NVT QM/MM dynamics in eight replicates. A time step of 0.5 fs was used. To this point, Cys spontaneously reprotonated in seven out of eight replicates with Ser168 in X-ray conformation and all replicates with AFm conformation.

### QM/MM Metadynamics

QM/MM well-tempered metadynamics simulations were performed for each of the four bound and four free models of TEVp.^63^ Two collective variables were used. The first one was defined as d(Cys151SG-H)–d(His46NE2-H). H here is either Cys151HG or His46HE2. The second CV was a torsion angle defined by atoms of Ser168: N–CA–CB–OG. The Gaussian potential with a height of 1 kJ/mol and a width of (0.02 nm, 0.25 rad.) was deposited every 100 steps. The bias factor was set to 12. Detailed Plumed input files can be found in the Supporting Information.

For each model, we initiated eight walkers starting from eight QM/MM equilibration replicates described earlier. Each walker ran for 150 ps with a 0.5 fs time step. Aggregate information from walkers was used to assess and confirm the convergence of simulations for each individual starting model (Figures S3 and S4). Aggregate information between different starting models was used to assess and confirm reproducibility (Figures S3 and S4) and build final free energy profiles. This way, for both peptide-bound and -free forms, 4.8 ns of simulations were aggregated. In each scenario, Tiwary reweighting^64^ was used to build profiles with the first 20 ps of a walker run discarded as the initial transient. The same scheme was utilized later to project profiles to different variables.

QM/MM calculations were carried out using the equipment of the shared research facilities of HPC computing resources at the Lomonosov Moscow State University.^65^

### MM Metadynamics

Prior to metadynamics, a 10 ns unrestrained NPT run was performed for each “protease-bound/free” combination starting from six independent restrained equilibration runs as described before. Since the starting bias for the driving variable was removed by the design of starting systems, these runs were made only to ensure the absence of any orthogonal processes that may influence the subsequent main production metadynamics run. We found that 10 ns equilibration is enough for this task. A single 2 μs run was performed for the TEVp–peptide complex, and six states after 1 μs were extracted, three for each Ser168 conformation similar to a general setup. For the X-ray conformation, frames from 1220, 1420, and 1780 ns were taken. For the AFm conformation, frames from 1300, 1600, and 1920 ns were taken (see the Results section). The comparison of free energy profiles after metadynamics runs starting from these states and ones taken after 10 ns showed no qualitative difference (Figure S5).

MM metadynamics for each system was performed by starting an independent run from each corresponding equilibration. Only one collective variable, Ser168 N–CA–CB–OG torsion, was used. The Gaussian potential with a height of 1 kJ/mol and a width of 0.25 radian was deposited every 1000 steps. The bias factor was set to 12. Simulations were run for 100 ns per replica with a 2 fs time step. Similar time scales and parameters were previously used in similar tasks with a driving variable expressed as a torsion angle.^66−68^ Profiles were built with Tiwary reweighting by discarding the first 20 ns as the initial transient. The mean profile constructed from six runs was used as a final estimate (Figure S6). Convergence was assessed by tracking the free energy difference between two minima corresponding to N–CA–CB–OG torsion being −180° (AFm conformation) and −70° (X-ray conformation, Figure S7).

### Phylogenetic Analysis

Phylogenetic tree building and ancestral sequence reconstruction were performed with FireProt-ASR.^69^ The tree was visualized in iTOL.^70^

## Results

### Modeling the Structure of the TEVp Michaelis Complex

**Figure 2:**
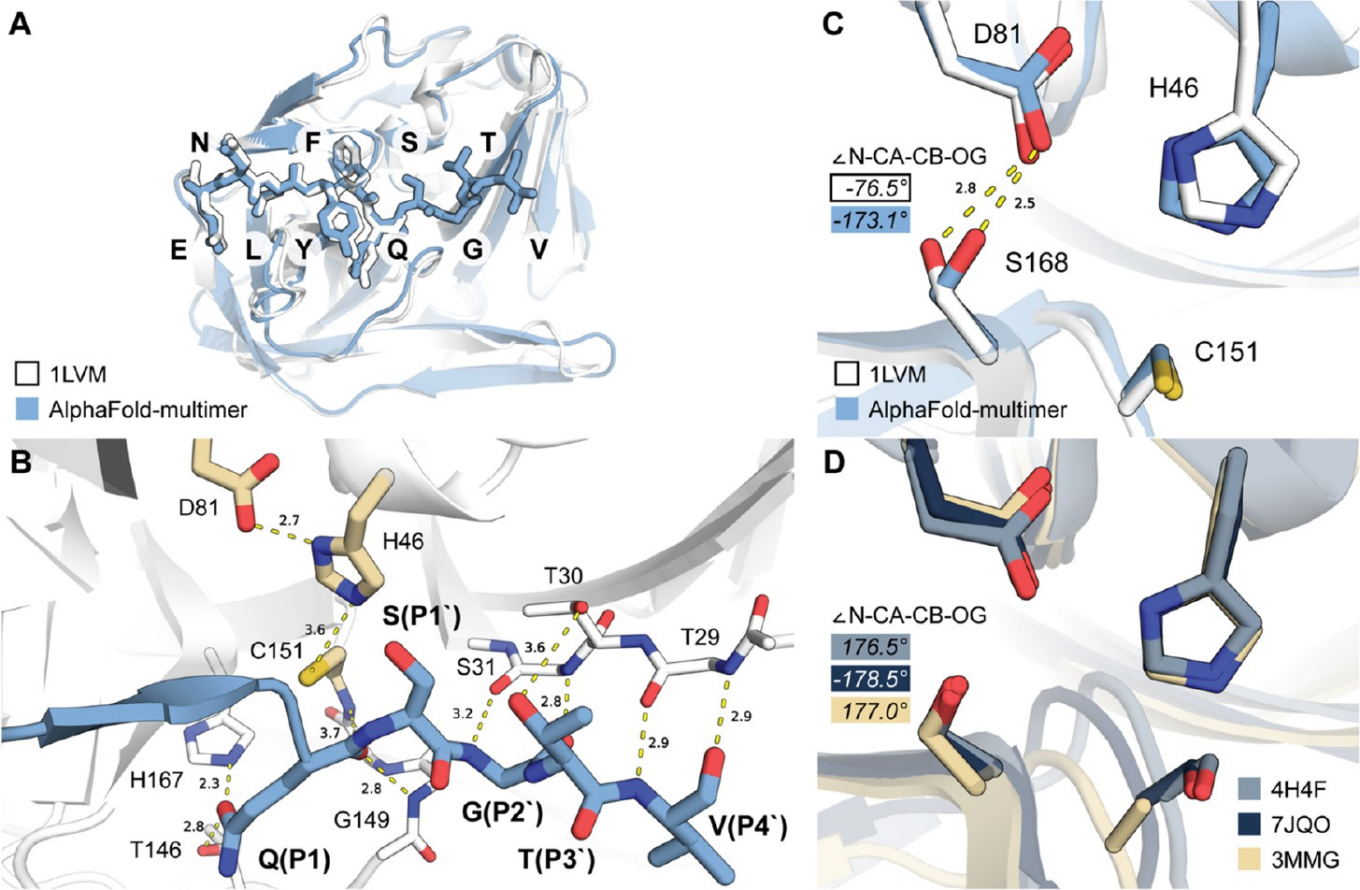
Features of a TEVp enzyme–substrate model constructed by AlphaFold2-multimer-v2. (A) Overview of the complex. (B) Active site structure and crucial enzyme–substrate interactions. (C) Differences in Ser168 conformation between X-ray and AlphaFold models of TEVp. (D) Ser168 conformation in structurally closest enzymes. 4H4F: chymotrypsin C, 7JQO: kallikrein 4, and 3MMG: tobacco vein mottling virus protease. The AlphaFold2-multimer model shown is a raw output (no relaxation) from the best-ranking model.

A starting point to discern the TEVp catalytic mechanism is a description of the structure of its complex with the substrate, that is, a Michaelis complex. Naturally, no such structure could be obtained by X-ray crystallography or CryoEM without making the enzyme inactive. A plausible complex may be constructed by protein–peptide docking, but this is a notoriously difficult problem with a high possibility of failure.^71^ On the other hand, AlphaFold was recently shown to produce high-quality models of protein–peptide complexes.^72^ This is why we started with the AlphaFold-multimer-v2 (AFm)-produced model of the actual active Michaelis complex. To assess its adequacy, we compared it to the X-ray structure of an active TEVp with the product peptide ENLYFQ (Figure 2). Positions of all residues present in the X-ray model were perfectly reconstructed by AFm (Figure 2A), and the binding pattern of P1′–P4′ residues corresponds well to what would be expected (Figure 2B). Peptide residues along the scissile bond were correctly positioned in the oxyanion hole with the leaving group nitrogen unoccluded to receive a proton from catalytic histidine further on (Figure 2B).

While the peptide pose was reconstructed well, we observed a deviation from the X-ray model in the structure of the enzyme itself. The conformation of Ser168 clearly differs between the two models (Figure 2C). In X-ray models, this residue faces inward, making H-bonds with both Asp81 (oxygen–oxygen distance 2.8 Å) and Thr180 (2.6 Å). N–CA– CB–OG torsio n in X-ray model 1LVM is −76.5°. In AFm models, Ser168 faces outward and H-bonds only Asp81 with an oxygen–oxygen distance of 2.5 Å and the associated N–CA–CB–OG torsion of −173.1°.

To gain support for either of these conformations, we searched for PA clan proteases closest to TEVp in terms of fold and came up with kallikrein 4 (KLK4), chymotrypsin C, and tobacco vein mottling virus (TVMV) protease. In all three enzymes, the conformation of corresponding Ser matches closely the one from the AFm model of TEVp. Moreover, we did not find any mention of the conformation like the one from TEVp X-ray models in the literature. Therefore, at this point, It was unclear whether there is an artifact in the X-ray model or if AFm is simply biased toward a more prominent conformation from PDB.^73,74^

Despite our primary focus being on the protonation state of catalytic Cys151, the influence of Ser168 conformation on it cannot be ruled out. Since Ser168 forms H-bonds with catalytic Asp81, their conformational landscapes may be linked. Changes in the relative positions of catalytic Asp81 and His46 may in their turn lead to alterations in His46 pKa, thus directly affecting the catalytic Cys151 protonation state. Thus, these two phenomena may be linked and should be studied together.

**Figure 3:**
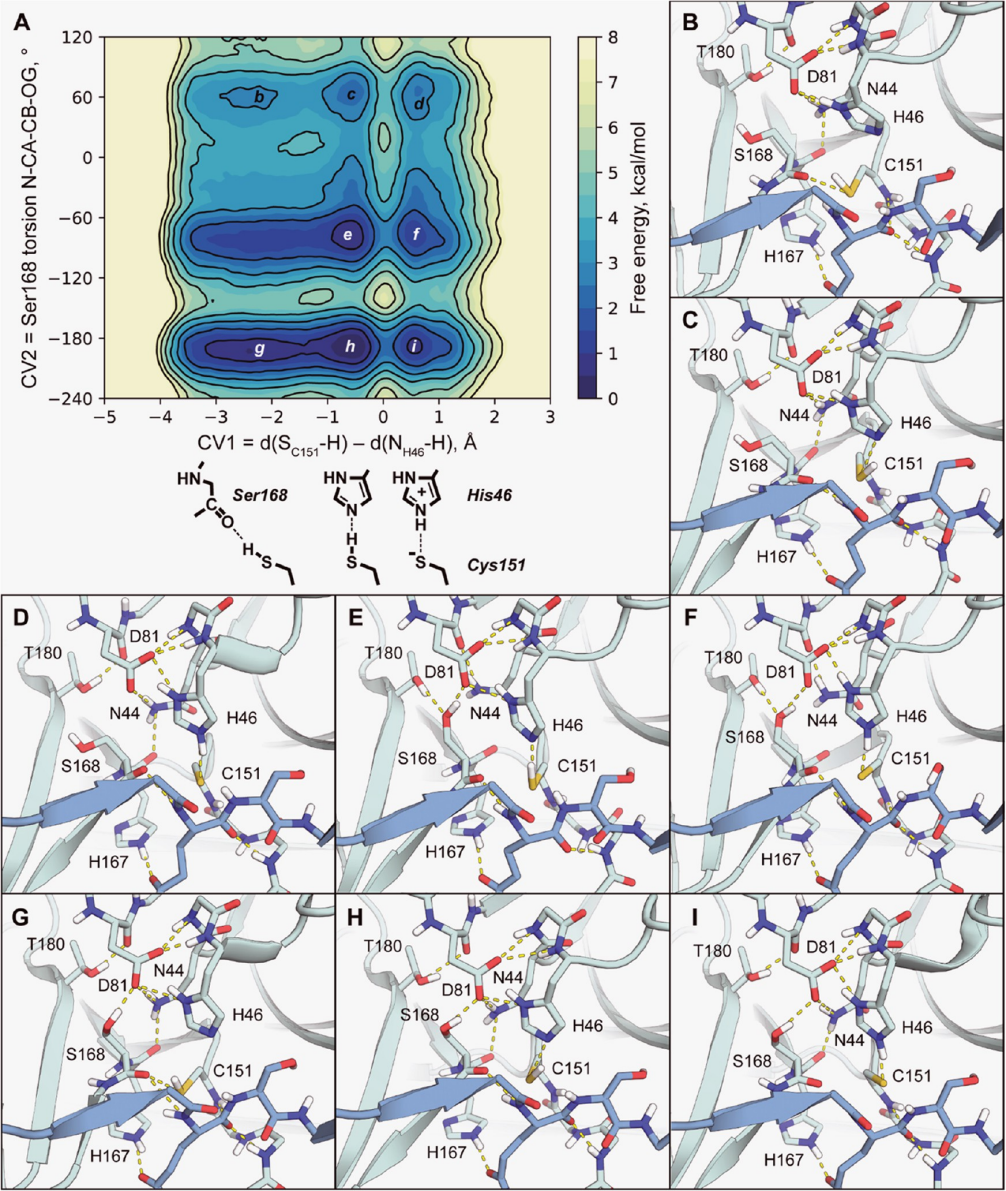
Influence of Ser168 conformation on the catalytic cysteine protonation state in the TEVp enzyme–substrate complex. (A) Free energy profile. One color level corresponds to the 0.5 kcal/mol step, and one outline to 1 kcal/mol. Letters b–i mark free energy minima that are further shown in detail. (B–I) Example frames for minima b–i from panel A, respectively. PDB files for these frames are available in the Supporting Information.

We performed a QM/MM metadynamics study to reconstruct the energy profile of two presumably linked processes—Cys deprotonation and Ser rotation along its CA–CB bond (Figure 3). We found that all three theoretically possible Ser conformations can occur in practice and interchange, although the two discussed previously are prevalent. X-ray Ser conformation in the immediate prereaction state is 0.6 kcal/mol less probable than the AFm one (Table 1 and Figure 3A,E,H). With any Ser conformation, the barrier of Cys deprotonation is under 3 kcal/mol, and the deprotonated state (Figure 3D,F,I) is 0.2–0.8 kcal/mol less probable than the protonated one (Table 1). Cys in the protonated state is highly mobile and capable of forming a stable self-inhibited state by donating an H-bond to the backbone oxygen of S168 (Figures 3B and4G). This state is especially prominent with Ser168 N–CA–CB–OG torsion values of approximately 180° (AFm conformation) and 60° (minor conformation).

**Figure 4:**
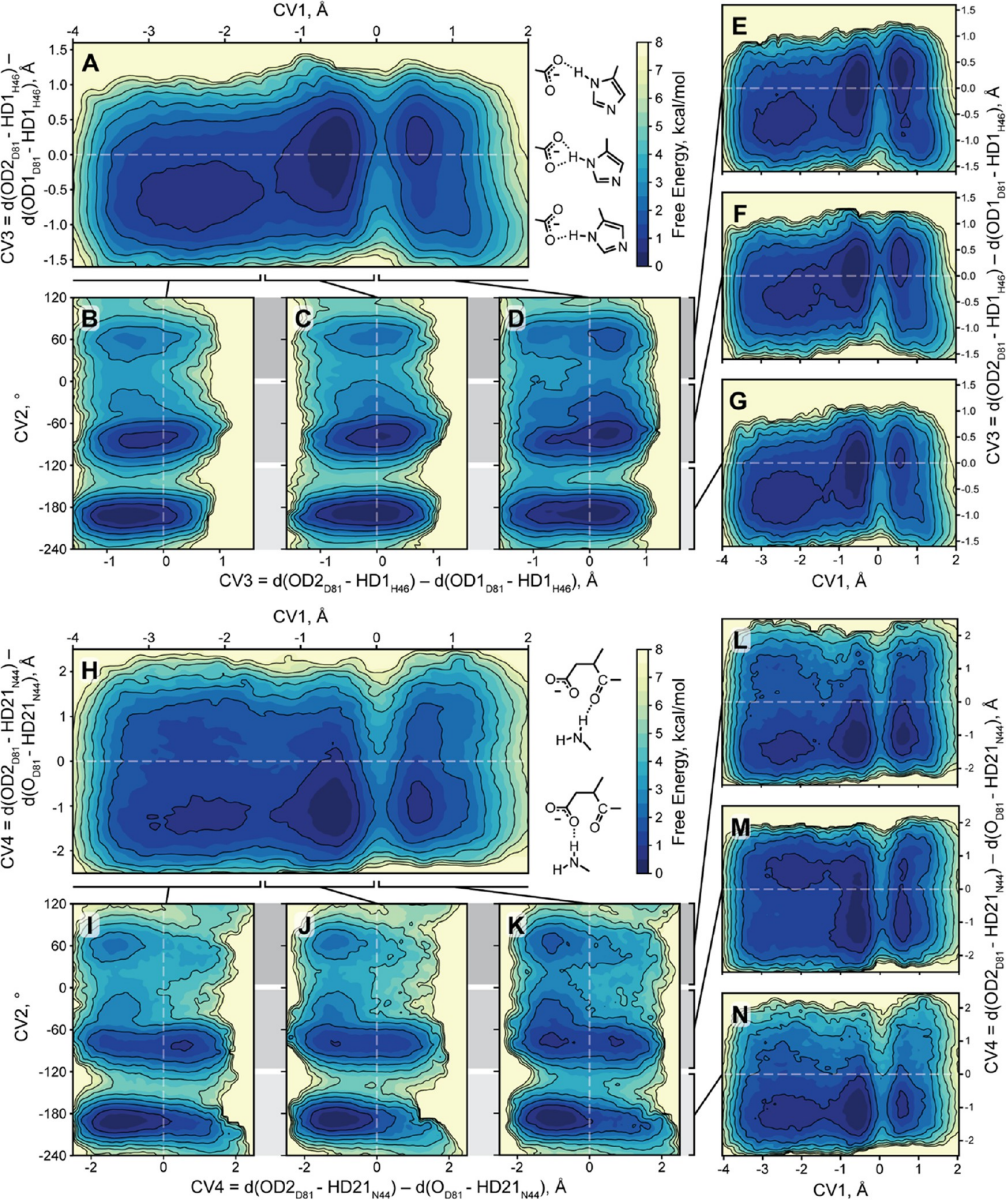
Changes in the active site structure along Cys151 deprotonation under the influence of Ser168 conformation. All are reweighted profiles. (A, E–G). Changes in the His46–Asp81 interaction geometry along the deprotonation process for all Ser168 conformations combined (A) and for a particular Ser168 conformation in isolation (E–G). (B–D) Dependence of the His46–Asp81 interaction geometry in particular stages of the deprotonation process on Ser168 conformation. (H, L–N). Changes in the Asn44 interaction pattern along the deprotonation process for all Ser168 conformations combined (H) and for a particular Ser168 conformation in isolation (L–N). (I–K). Dependence of the Asn44 interaction pattern in particular stages of the deprotonation process on Ser168 conformation. One color level at free energy profiles corresponds to the 0.5 kcal/mol step, and one outline to 1 kcal/mol.

**Table 1:** Parameters of Free Energy Minima from Figure 3A

state	b	c	d	e	f	g	h	i
G, kcal/mol	2.8 ± 0.1	2.2 ± 0.2	2.4 ± 0.2	0.6 ± 0.2	1.1 ± 0.3	0.5 ± 0.2	0 ± 0.1	0.8 ± 0.3
CV1, Å	–2.3	–0.6	0.6	–0.6	0.6	–2.4	–0.6	0.6
CV2, °	57	62	65	–79	–79	166	175	172

Cys deprotonation is associated with subtle conformational rearrangements in the active center (Figure 4), and these events depend on Ser168 conformation. In the self-inhibited state, catalytic His46 exclusively forms H-bonds with the catalytic Asp81 Oδ2 oxygen, the one closer to Ser168 (Figures 3B,G and4A,B,E–G). It is especially so for AFm conformation (Figure 4B). At the prereaction state, His mostly forms a bifurcated H-bond with both Asp oxygens (Figure 4C). This H-bond is symmetrical in minor Ser conformation, skewed to Oδ2 in AFm conformation and to Oδ1 in X-ray conformation. In a deprotonated state, His shifts to mostly form an H-bond to Oδ1 oxygen (Figure 4D). This is most clearly pronounced for minor Ser conformation and less so for AFm conformation. Interestingly, while at the deprotonated state, a transition between AFm and X-ray conformations requires His to shift back to form an exclusive H-bond to Oδ2 (Figure 4D). With all three Ser conformations, deprotonation occurs more preferably with His switched to Oδ1 (Figure 4A,E–G).

A more dramatic influence of Ser168 conformation is on Asn44 (Figure 4H–N). This residue is also a part of the active site and can form H-bond with either catalytic Asp81 Oδ2 (Figure 3B) or its backbone oxygen (Figure 3F). In minor and AFm Ser168 conformations, it is generally the former (Figure 4L,N), while in X-ray conformation, its behavior changes along the deprotonation process (Figure 4M). In the self-inhibited state, Asn44 interacts with backbone oxygen, while in the prereaction state, the balance shifts to donate an H-bond to Asp81. It makes evolutionary sense, since Cys deprotonation preferably proceeds with this interaction pattern of Asn44 (Figure 4M). In the deprotonated state, an equilibrium of Asn44 conformations is present (Figure 4K,M).

**Figure 5:**
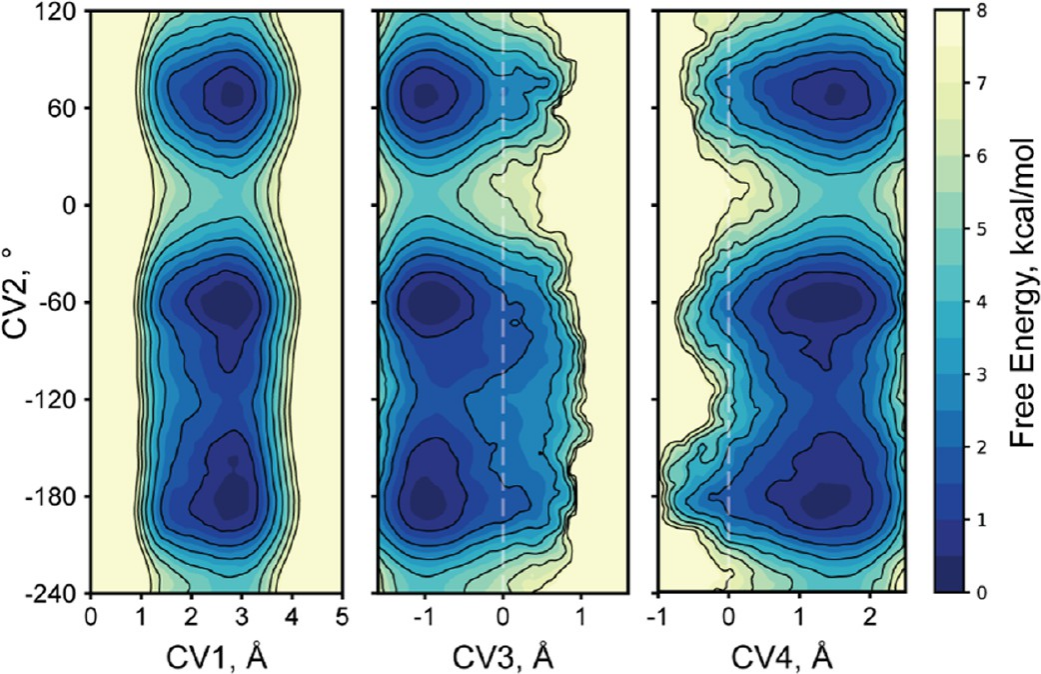
Lack of variation in the active site structure of free TEVp. From left to right: strict deprotonation of Cys151, strict His46–Asp81 Oδ2 H-bonding, strict Asn44–backbone interaction. One color level corresponds to the 0.5 kcal/mol step, and one outline to 1 kcal/mol.

The situation changes in the absence of a bound peptide. In this state, Cys is strictly deprotonated, His46 donates an H-bond to Oδ2 oxygen atom of Asp81, and Asn44 interacts with Asp81 backbone oxygen (Figure 5). Peptide binding, therefore, plays a major role in bringing Cys and His close to each other and shielding them from water. Without a peptide, the transition between AFm and X-ray conformations also becomes much easier. There are no differences in the system′s behavior dependent on Ser168 conformation.

**Figure 6:**
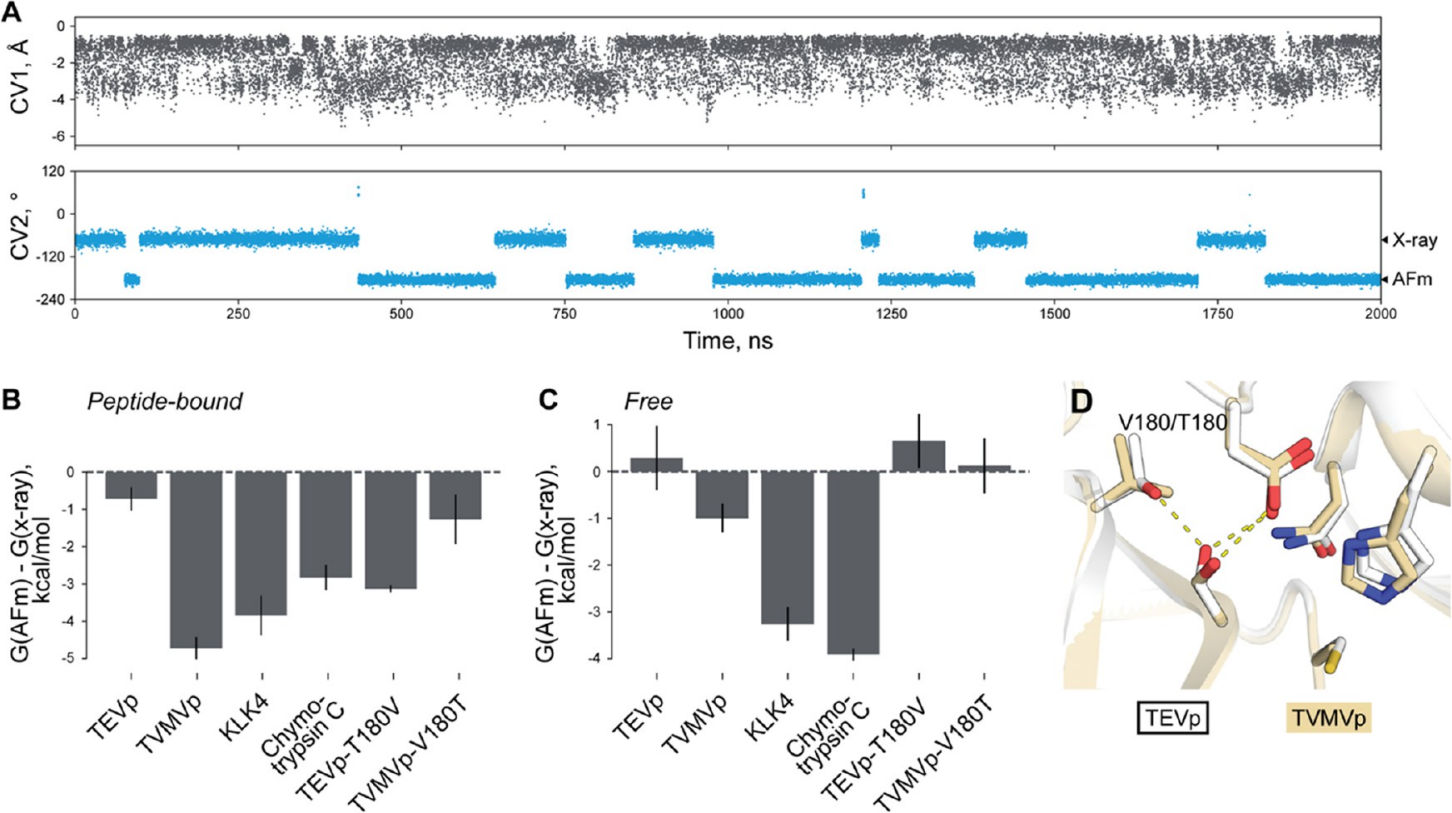
Varying behavior of Ser168 and corresponding residues in a number of PA clan proteases and their variants. (A) Cys151 and Ser168 of TEVp are highly mobile. Two major conformations for Ser168, X-ray, and AFm are highlighted on a torsion axis. (B) Preference for AFm conformation in a peptide-bound form of TEVp, related enzymes, and mutant variants. (C) Preference for AFm conformation in a free form of TEVp, related enzymes, and mutant variants. (D) Influence of the identity of position 180 on Ser168 conformation in TEVp and TVMVp.

To this point, using QM/MM metadynamics, we discovered that in the structure of TEVp at the Michaelis complex, two Ser168 conformations are almost equally possible and that the catalytic Cys151 is highly mobile. The same was also found to be true in a 2 μs MM run (Figure 6A). Such a conclusion could not be reached based on the analysis of available X-ray models alone. It is therefore possible that analogous Ser residues in other PA clan proteases can also be highly dynamic and feature such a stable alternative state. To explore this possibility, we performed a metadynamics run on both peptide-bound and -free models of previously mentioned proteases: KLK4, chymotrypsin C, and TVMVp. To be able to compare several systems, we were forced to use pure MM treatment. By comparing data for TEVp, we concluded that the MM description is capable of correctly ranking stable states by their energy (Figure S8). This allows for drawing only qualitative conclusions on the minima. MM treatment is clearly much stiffer, and therefore, barrier heights do not match.

Neither of these three proteases displayed the same Ser conformational pattern in the peptide-bound complex as TEVp, making it indeed a unique feature of this enzyme (Figure 6B,C). There was a pronounced difference even compared to another cysteine protease in the set, TVMVp, which is the closest TEVp homologue with a known X-ray structure. In a peptide-bound form, in both proteases, Ser168 in the X-ray conformation competes with Asn44 for providing an H-bond to Asp81 Oδ2. However, in TEVp, this conformation is stabilized by the H-bond from Thr180 that cannot be formed in TVMVp, since it has Val in this position (Figure 6D). The difference between the two cysteine proteases is however diminished when the free models are compared (Figure 6C). In TVMVp without the peptide, Asn44 interacts with Asp81 backbone oxygen instead of Oδ2, which frees Ser168 from competing with it.

Virtually substituting Thr180 for Val in peptide-bound TEVp makes it very similar to TVMVp in terms of Ser behavior (Figure 6B). Likewise, restoring Thr in TVMVp brings it closer to TEVp and further from two serine PA proteases under consideration (Figure 6B). It should be noted that KLK4 and chymotrypsin C originally harbor Thr corresponding to TEVp Thr180, and nothing competes with the Ser168 analogue for aspartate. Yet, their behavior is different possibly due to the exposure of the Ser168 analogue to water even in the peptide-bound form (Figure S9).

We performed phylogenetic analysis and ancestral sequence reconstruction of TEVp homologues that are viral Cys PA clan proteases. The tree topology correlates well with the identity of residue 180 (Figure S10). Threonine at this position is the oldest variant (Thr probability in the ultimate ancestor at 79%), and clades with predominant Ala180 and Val180 emerge. The biggest Val180 clade is strongly associated with Ala at position 168 instead of Ser. Hence, any influence of Ser conformation on other parts of the active site is not necessary for proteolytic activity arising from implementing a Cys nucleophile in a chymotrypsin-like fold.

TEVp and TVMVp are indeed very closely related and belong to a mostly Thr180 clade. Val in TVMVp is a novel feature, which correlates well with our virtual mutagenesis results. It however remains unclear whether Ser mobility in TEVp has any connection to function at all or is just a tolerable neutral feature. To arrive at a definite conclusion, an insight into the full reaction process, both acylation and deacylation stages, must be gained in further work.

## Discussion

In this work, we took an unconventional approach to construct a protease–substrate complex by utilizing the capabilities of AlphaFold-multimer. No assessment of AFm ability to predict protease–peptide complexes was previously made. Here, we show that it performed near perfect for a range of PA clan proteases.

It is intriguing how and why it achieved this result. To fold a protein, AlphaFold utilizes information on residue coevolution that comes from multiple sequence alignment (MSA) embeddings and is contained in an MSA representation.^75^ It is then used to construct and update a pair representation. This second representation contains information on residue–residue distances and is processed to ensure triangle inequality. Processed pair representation is then used to update MSA representation in a new update round. AlphaFold-multimer is a follow-up system based on the ideas of AlphaFold designed to predict the structures of multichain protein complexes. As inputs, AlphaFold-multimer utilizes paired alignments constructed for each individual chain in the assignment.^30^ Pairing is performed based on phylogenetics using the UniProt species annotation.

Our use of AlphaFold-multimer is, however, different in a way that no alignment is constructed for a short substrate peptide. The same is true for a previously proposed way of using AlphaFold for peptide–protein docking.^72^ By attaching a peptide to a protein via a polyglycine linker, researchers were able to obtain good-quality complexes. No alignment was built for polyglycine and peptide regions. In both ways, protein–peptide coevolution is still taken into account normally, since peptide′s sequence is effectively a degenerate alignment with no variation. Both methods work since the physicochemical interactions that drive protein folding and protein–peptide interactions are the same.^76^ Protease binding sites′ evolve to better recognize alien substrate loops and cognate substrate loops coevolve with their respective proteases.^77−79^

**Figure 7:**
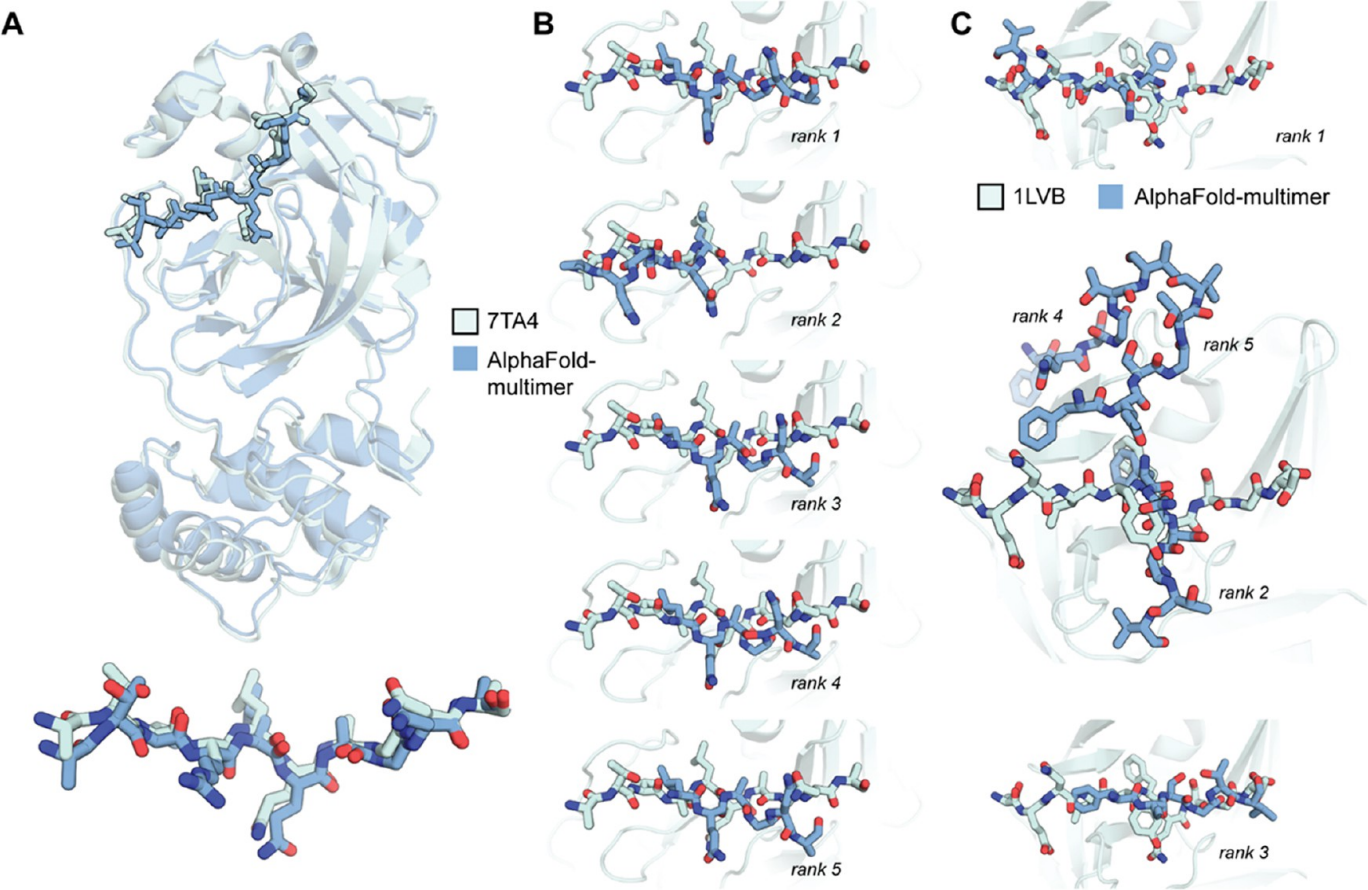
AlphaFold-multimer-v2 performance on a complex absent in the training data and with truncated substrate peptides. (A) AlphaFold-multimer achieves good quality in constructing a complex between the SARS-Cov-2 main protease (MPro) and its substrate peptide ATVRLQAGNA. The best-ranked model is presented. (B, C) AlphaFold-multimer struggles to place a substrate peptide when the main interacting region is absent. (B) Predictions for the SARS-Cov-2 main protease and the peptide LQAGNA. (C) Predictions for TEVp and the peptide FQSGTV. AlphaFold models are shown in blue, X-ray references in aqua, 7TA4 for SARS-Cov-2 MPro (A, B), and 1LVB for TEVp (C).

In the paper describing the approach with a polyglycine linker, researchers conclude that the good quality of complexes does not arise from just memorization. We, however, decided to verify this conclusion for our method and application field. AlphaFold-multimer was trained on PDB structures with a maximum release date of 2018-04-30. First-ever complexes between Sars-Cov-2 MPro (or 3CLPro), a PA clan Cys protease, and its substrates were resolved in 2020.^80^ AlphaFold-multimer was able to produce a high-quality prediction nevertheless (Figure 7A).

The absence of a peptide alignment means that no information on peptide conformation comes from the peptide sequence itself. Therefore, a protein here completely guides the folding of a substrate peptide, which is in accordance with how it happens in nature.^81^ Substrate peptides in PA clan proteases come from unstructured loops. A β-sheet is formed between a protease and a region of the substrate loop downstream of the scissile bond. We speculate that no adequate complex can be built by AlphaFold when this region is not provided. Our runs on TEV and Sars-Cov-2 MPro with truncated peptides support our hypothesis (Figure 7B,C).

A further assessment of a broader selection of various proteases is needed to reach a generalized conclusion on whether AlphaFold-multimer can be broadly recommended in similar studies on protease–substrate complex structure prediction. However, its overall decent ability to perform protein–peptide docking^72^ combined with data presented herein makes it possible to speculate that AlphaFold-multimer might be a general solution to such a task. Once its ability to predict Michaelis complexes of proteases is proven, it may even be used to screen for protease decoys against a particular substrate peptide for further fine-tuning using methods of computational protein design.^82,83^

AlphaFold also turned out to be right about the possibility of another Ser168 conformation. However, all five models produced the same result, thus giving no hint on the possibility of X-ray conformation and strengthening assumptions that AF is biased toward available structural data in PDB.^73,74^ Thus, without X-ray structures, starting from AF models, we could have easily overlooked this alternative state. Could this limitation be overcome? We took inspiration from recent works, suggesting that trimming the alignment may result in different conformations,^84^ and indeed obtained both Ser forms discussed in this work (Figure S11). This shows that AF may be a powerful tool for providing hypotheses on cryptic but inherent conformational switches in protein structures not immediately obvious from X-ray models.

**Figure 8:**
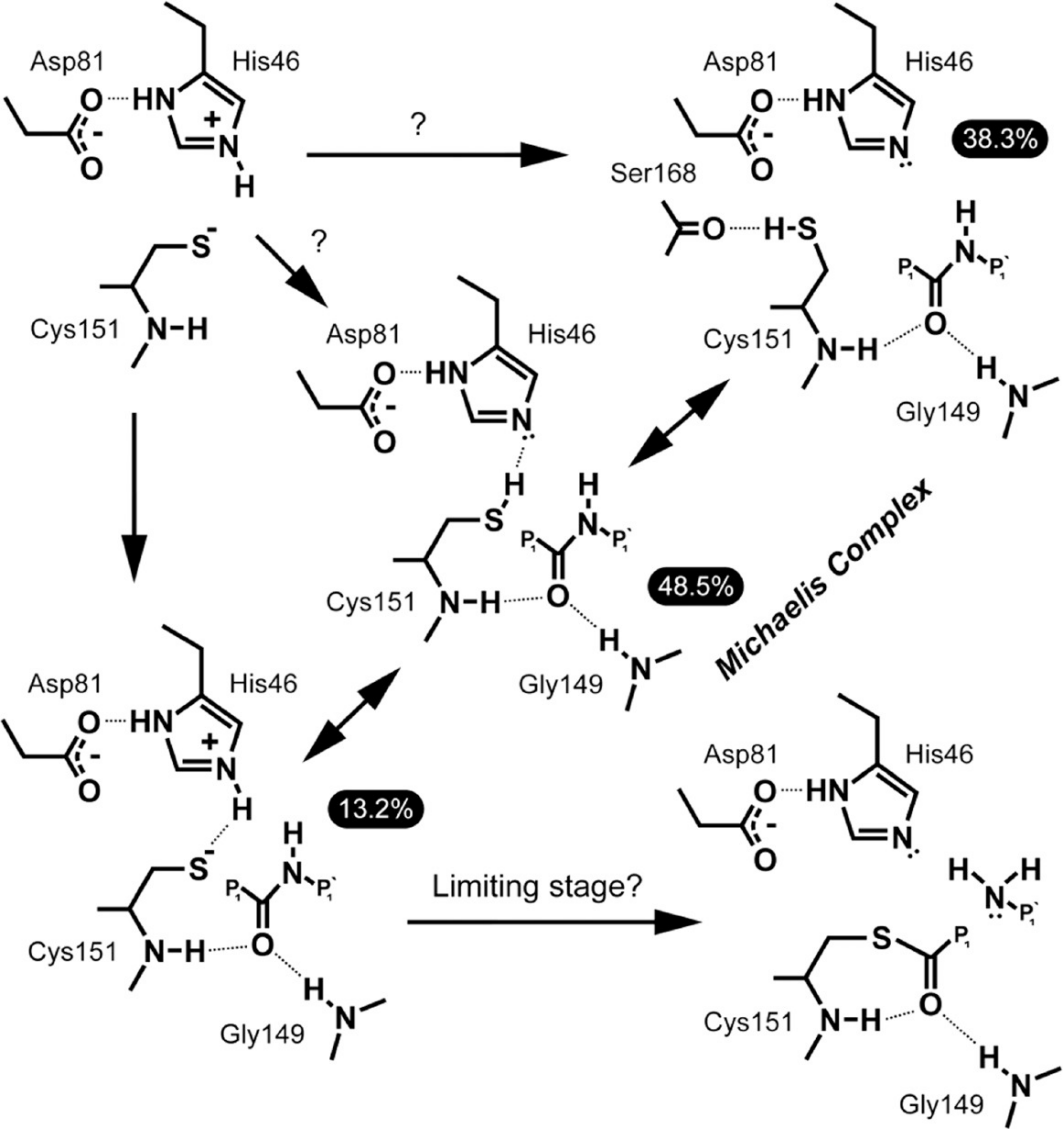
Catalytic mechanism of the acylation stage of TEVp proposed based on results of this work. Sub-states within the Michaelis complex are shown on a gray background with relative populations highlighted.

Since the Cys deprotonated state turned out to be quite possible on its own without anything happening to the substrate, the acylation stage of the proteolysis reaction must proceed similarly to the papain-like mechanism (Figure 8). It is believed to normally feature only one stage of highly concerted simultaneous breakage of C–N(pept) and N(His)–H bonds and formation of S–C and N(peptide)–H bonds.^26−28^ Due to the complications of orchestrating such a process, the associated barrier is high and it is usually a limiting stage. TEVp, however, faces another obstacle on the path to becoming a fast enzyme. As we show here, it demonstrates a tendency to fall back into two states with protonated Cys that are effectively inhibited forms of the enzyme. Our QM/MM calculations show that only 13.2% of configurations in the Michaelis complex are productive. Careful considerations of the exact populations of substrates within what may be otherwise considered to be a single state are necessary to construct correct kinetic schemes. This, in turn, may lead to revolutionary new findings on the enzyme′s efficiency as was recently shown for the staphylokinase–plasmin complex.^85^

TEVp is not unique in the ability of its nucleophile to lose a hydrogen bond to the catalytic histidine. The same behavior was proposed to be the cause behind the existence of the “slow” form of thrombin.^86^ Allosteric activation by a Na^+^ ion turns it into a 10 times more active “fast” form by shifting the populations of conformations of the catalytic nucleophile.^86,87^ Therefore, a possible strategy to enhance TEVp and similar enzymes would be to discourage such a process or to even lock Cys in a constantly deprotonated state.

While Cys reprotonation is likely a manifestation of a fold being underoptimized for such a nucleophile, the surroundings of active sites of Cys PA clan proteases are clearly different from their Ser counterparts with Asn44 being an obvious example. Thus, some degree of optimization was nevertheless achieved, and it is an intriguing further direction to correlate it with concrete features. Inspiration might be taken from studying the opposite process—rolling back to a Ser nucleophile. A series of such TEVp mutants, leading to improved activity in Cys151Ser, was previously obtained.^22^ Overall, the following questions emerge: is there even space to further fine-tune the chymotrypsin-like fold for cysteine as a nucleophile, and why was serine swept out of natural selection in the first place? It is possible that first came crucial alterations in the fold itself and cysteine acted as a compensatory substitution.

TEVp and TVMVp are not the only proteases featuring a cysteine nucleophile in a chymotrypsin-like fold. It is a quite common feature among virus proteases including 3CLpro from SARS-CoV-2 mentioned earlier. It may be tempting to transfer results presented herein for enzyme–substrate complexes on other such proteases. However, caution is advised, since many of them deviate significantly in terms of how acid function is implemented (Figure S12). Differences such as Glu instead of Asp or water occupying its place may significantly alter histidine basicity, and the extent of such alterations should be better investigated in a separate study. On the other hand, for the free form of such enzymes, evidence mounts suggesting the zwitterionic state of the catalytic site. Neutron crystallography performed for SARS-Cov-2 3CLpro corresponds well to the data we acquired herein.^88^

## Conclusions

The TEV protease is the most extensively studied member of PA clan proteases with cysteine as a nucleophile. We expand on previous knowledge by providing a detailed insight into the equilibrium of states of the TEVp enzyme–substrate complex as well as the free enzyme. We perform enhanced sampling QM/MM calculations to show, in agreement with previous works, that catalytic cysteine is deprotonated if the substrate peptide is not present. Substrate binding, however, turned out to induce the possibility of cysteine protonation. The barrier between two protonation states is small, thus prompting rapid interconversion of states, with the protonated state slightly more stable than the deprotonated one. Cysteine deprotonation does not result in a spontaneous attack on the substrate carbon atom. We, therefore, suggest that starting from this deprotonated state, a papain-like catalytic mechanism might be valid for TEVp.

We demonstrate that protonated catalytic cysteine is highly mobile and may switch its H-bonding partner from catalytic histidine to the serine nearby. This state is effectively a self-inhibited state away from the proposed reaction path. The existence of this state is likely an outcome of under-optimization of preferentially serine triad-harboring folds for a nucleophile switch. It remains to be investigated whether this under-optimization is an apex of what can be achieved without complete fold change or if there is still space for minor improvement.

We also uncovered the existence of two almost equally possible conformations of the same serine that TEVp catalytic cysteine may form an H-bond to. The analysis of structurally similar proteases, both serine and cysteine, as well as homologues, indicated that this is not a general feature of cysteine PA clan proteases and may be an individual feature of TEVp. We showed that these conformations affect the structure of the active site as it proceeds with cysteine deprotonation, however, with no meaningful effect on the possibility of the process or populations of states. Such effects may manifest themselves during later stages of the reaction.

Through the calculation and analysis performed in this work, we uncover new details about how cysteine proteases may perform their functions and propose a plausible strategy to improve the activity of TEVp and similar enzymes. Beyond that, we demonstrated a surprisingly sound ability of AlphaFold-multimer in constructing meaningful complexes of proteases with their respective peptide substrates. This work presents a case highlighting that a combination of different modeling techniques can facilitate advances in the understanding of structure–function relationships of enzymes.

## Supporting Information Available

Fold and active site differences between PA proteases and papain (Figure S1); QM region composition (Figure S2); convergence and reproducibility in QM/MM metadynamics runs (Figures S3); comparison of MetaD results when initiating from six independent 10 ns equilibration runs and from six frames taken after the 1 μs of a 2 μs run (Figure S4); comparison of QM/MM and MM reconstructions of the Ser168 torsion free energy profile (Figure S5); free energy profiles of Ser CA–CB rotation from MM runs (Figure S6); convergence of MM metadynamics runs tracked by the free energy difference between Ser states (Figure S7); comparison of free energy profiles of Ser168 rotation obtained from QM/MM and pure MM calculations (Figure S8); hydration in the active site of serine PA clan proteases in a peptide-bound form (Figure S9); phylogenetic tree of viral cysteine PA clan proteases (Figure S10); AlphaFold2 models from trimmed alignments (Figure S11); and showcase of different acid identities and architecture across distant viral PA clan cysteine proteases (Figure S12).
Supplemental Data including example states from QM/MM metadynamics runs, AlphaFold2-multimer-produced models, and inputted multiple sequence alignments, input files for PLUMED, and FireProt-ASR output are deposited on Zenodo and can be accessed by DOI: 10.5281/zenodo.7093045 (PDF)

## References

[ref1] López-Otín C.; Bond J. S. Proteases: Multifunctional Enzymes in Life and Disease. J. Biol. Chem. 2008, 283, 30433–30437. 10.1074/jbc.R800035200. 18650443 PMC2576539

[ref2] Cesaratto F.; Burrone O. R.; Petris G. Tobacco Etch Virus Protease: A Shortcut across Biotechnologies. J. Biotechnol. 2016, 231, 239–249. 10.1016/j.jbiotec.2016.06.012. 27312702

[ref3] Raran-Kurussi S.; Cherry S.; Zhang D.; Waugh D. S. Removal of Affinity Tags with TEV Protease. Methods Mol. Biol. 2017, 1586, 221–230. 28470608 10.1007/978-1-4939-6887-9_14 PMC7974378

[ref4] Wang W.; Wildes C. P.; Pattarabanjird T.; Sanchez M. I.; Glober G. F.; Matthews G. A.; Tye K. M.; Ting A. Y. A Light- and Calcium-Gated Transcription Factor for Imaging and Manipulating Activated Neurons. Nat. Biotechnol. 2017, 35, 864–871. 10.1038/nbt.3909. 28650461 PMC5595644

[ref5] Barnea G.; Strapps W.; Herrada G.; Berman Y.; Ong J.; Kloss B.; Axel R.; Lee K. J. The Genetic Design of Signaling Cascades to Record Receptor Activation. Proc. Natl. Acad. Sci. U. S. A. 2008, 105, 64–69. 10.1073/pnas.0710487105. 18165312 PMC2224232

[ref6] Gao X. J.; Chong L. S.; Kim M. S.; Elowitz M. B. Programmable Protein Circuits in Living Cells. Science 2018, 361, 1252–1258. 10.1126/science.aat5062. 30237357 PMC7176481

[ref7] Fink T.; Lonzarić J.; Praznik A.; Plaper T.; Merljak E.; Leben K.; Jerala N.; Lebar T.; Strmšek Ž.; Lapenta F.; Benčina M.; Jerala R. Design of Fast Proteolysis-Based Signaling and Logic Circuits in Mammalian Cells. Nat. Chem. Biol. 2019, 15, 115–122. 10.1038/s41589-018-0181-6. 30531965 PMC7069760

[ref8] Mohammadian H.; Mahnam K.; Sadeghi H. M.; Ganjalikhany M. R.; Akbari V. Rational Design of a New Mutant of Tobacco Etch Virus Protease in Order to Increase the Solubility. Res. Pharm. Sci. 2020, 15, 164–173. 10.4103/1735-5362.283816. 32582356 PMC7306250

[ref9] Nautiyal K.; Kuroda Y. A SEP Tag Enhances the Expression, Solubility and Yield of Recombinant TEV Protease without Altering Its Activity. New Biotechnol. 2018, 42, 77–84. 10.1016/j.nbt.2018.02.006. 29448030

[ref10] Nam H.; Hwang B. J.; Choi D.-Y.; Shin S.; Choi M. Tobacco Etch Virus (TEV) Protease with Multiple Mutations to Improve Solubility and Reduce Self-Cleavage Exhibits Enhanced Enzymatic Activity. FEBS Open Bio 2020, 10, 619–626. 10.1002/2211-5463.12828. PMC7137792 32129006

[ref11] Bayar E.; Ren Y.; Chen Y.; Hu Y.; Zhang S.; Yu X.; Fan J. Construction, Investigation and Application of TEV Protease Variants with Improved Oxidative Stability. J. Microbiol. Biotechnol. 2021, 31, 1732–1740. 10.4014/jmb.2106.06075. 34528919 PMC9705859

[ref12] Norris J. L.; Patel T.; Dasari A. K. R.; Cope T. A.; Lim K. H.; Hughes R. M. Covalent and Non-Covalent Strategies for the Immobilization of Tobacco Etch Virus Protease (TEVp) on Superparamagnetic Nanoparticles. J. Biotechnol. 2020, 322, 1–9. 10.1016/j.jbiotec.2020.06.021. 32619644

[ref13] Denard C. A.; Paresi C.; Yaghi R.; McGinnis N.; Bennett Z.; Yi L.; Georgiou G.; Iverson B. L. YESS 2.0, a Tunable Platform for Enzyme Evolution, Yields Highly Active TEV Protease Variants. ACS Synth. Biol. 2021, 10, 63–71. 10.1021/acssynbio.0c00452. 33401904

[ref14] Sanchez M. I.; Ting A. Y. Directed Evolution Improves the Catalytic Efficiency of TEV Protease. Nat. Methods 2020, 17, 167–174. 10.1038/s41592-019-0665-7. 31819267 PMC7004888

[ref15] Amrein B. A.; Steffen-Munsberg F.; Szeler I.; Purg M.; Kulkarni Y.; Kamerlin S. C. L. Computer-Aided Directed Evolution of Enzymes. IUCrJ 2017, 4, 50–64. 10.1107/S2052252516018017. PMC5331465 28250941

[ref16] Wei R.; von Haugwitz G.; Pfaff L.; Mican J.; Badenhorst C. P. S.; Liu W.; Weber G.; Austin H. P.; Bednar D.; Damborsky J.; Bornscheuer U. T. Mechanism-Based Design of Efficient PET Hydrolases. ACS Catal. 2022, 12, 3382–3396. 10.1021/acscatal.1c05856. 35368328 PMC8939324

[ref17] Mokrushina Y. A.; Golovin A. V.; Smirnov I. V.; Chatziefthimiou S. D.; Stepanova A. V.; Bobik T. V.; Zalevsky A. O.; Zlobin A. S.; Konovalov K. A.; Terekhov S. S.; Stepanov A. V.; Pipiya S. O.; Shamborant O. G.; Round E.; Belogurov A. A. Jr.; Bourenkov G.; Makarov A. A.; Wilmanns M.; Xie J.; Blackburn G. M.; Gabibov A. G.; Lerner R. A. Multiscale Computation Delivers Organophosphorus Reactivity and Stereoselectivity to Immunoglobulin Scavengers. Proc. Natl. Acad. Sci. U.S.A. 2020, 117, 22841–22848. 10.1073/pnas.2010317117. 32859757 PMC7502716

[ref18] Tian Y.; Xu Z.; Huang X.; Zhu Y. Computational Design to Improve Catalytic Activity of Cephalosporin C Acylase from Pseudomonas Strain N176. RSC Adv. 2017, 7, 30370–30375. 10.1039/C7RA04597B.

[ref19] Risso V. A.; Romero-Rivera A.; Gutierrez-Rus L. I.; Ortega-Muñoz M.; Santoyo-Gonzalez F.; Gavira J. A.; Sanchez-Ruiz J. M.; Kamerlin S. C. L. Enhancing a Enzyme Activity by Computationally-Focused Ultra-Low-Throughput Screening. Chem. Sci. 2020, 11, 6134–6148. 10.1039/D0SC01935F. 32832059 PMC7407621

[ref20] Li R.; Wijma H. J.; Song L.; Cui Y.; Otzen M.; Tian Y.′e.; Du J.; Li T.; Niu D.; Chen Y.; Feng J.; Han J.; Chen H.; Tao Y.; Janssen D. B.; Wu B. Computational Redesign of Enzymes for Regio- and Enantioselective Hydroamination. Nat. Chem. Biol. 2018, 14, 664–670. 10.1038/s41589-018-0053-0. 29785057

[ref21] Wolf C.; Siegel J. B.; Tinberg C.; Camarca A.; Gianfrani C.; Paski S.; Guan R.; Montelione G.; Baker D.; Pultz I. S. Engineering of Kuma030: A Gliadin Peptidase That Rapidly Degrades Immunogenic Gliadin Peptides in Gastric Conditions. J. Am. Chem. Soc. 2015, 137, 13106–13113. 10.1021/jacs.5b08325. 26374198 PMC4958374

[ref22] Shafee T.; Gatti-Lafranconi P.; Minter R.; Hollfelder F. Handicap-Recover Evolution Leads to a Chemically Versatile, Nucleophile-Permissive Protease. Chembiochem 2015, 16, 1866–1869. 10.1002/cbic.201500295. 26097079 PMC4576821

[ref23] Elsässer B.; Goettig P. Mechanisms of Proteolytic Enzymes and Their Inhibition in QM/MM Studies. Int. J. Mol. Sci. 2021, 22, 3232 10.3390/ijms22063232. 33810118 PMC8004986

[ref24] Zlobin A.; Ermidis A.-P.; Maslova V.; Belyaeva J.; Golovin A. Exploiting Structural Constraints of Proteolytic Catalytic Triads for Fast Supercomputer Scaffold Probing in Enzyme Design Studies. In Communications in Computer and Information Science; Springer International Publishing: Cham, 2021; pp 58–72.

[ref25] Cstorer A.; Ménard R. [33] Catalytic Mechanism in Papain Family of Cysteine Peptidases. In Methods in Enzymology; Methods in Enzymology; Elsevier, 1994; pp 486–500. 10.1016/0076-6879(94)44035-2 7845227

[ref26] Fekete A.; Komáromi I. Modeling the Archetype Cysteine Protease Reaction Using Dispersion Corrected Density Functional Methods in ONIOM-Type Hybrid QM/MM Calculations; the Proteolytic Reaction of Papain. Phys. Chem. Chem. Phys. 2016, 18, 32847–32861. 10.1039/C6CP06869C. 27883128

[ref27] Han W. G.; Tajkhorshid E.; Suhai S. QM/MM Study of the Active Site of Free Papain and of the NMA-Papain Complex. J. Biomol. Struct. Dyn. 1999, 16, 1019–1032. 10.1080/07391102.1999.10508311. 10333172

[ref28] Wei D.; Huang X.; Liu J.; Tang M.; Zhan C.-G. Reaction Pathway and Free Energy Profile for Papain-Catalyzed Hydrolysis of N-Acetyl-Phe-Gly 4-Nitroanilide. Biochemistry 2013, 52, 5145–5154. 10.1021/bi400629r. 23862626 PMC3770148

[ref29] Huggins D. J.; Biggin P. C.; Dämgen M. A.; Essex J. W.; Harris S. A.; Henchman R. H.; Khalid S.; Kuzmanic A.; Laughton C. A.; Michel J.; Mulholland A. J.; Rosta E.; Sansom M. S. P.; van der Kamp M. W. Biomolecular Simulations: From Dynamics and Mechanisms to Computational Assays of Biological Activity. Wiley Interdiscip. Rev.: Comput. Mol. Sci. 2019, 9, e1393 10.1002/wcms.1393.

[ref30] Evans R.; O’Neill M.; Pritzel A.; Antropova N.; Senior A.; Green T.; Žídek A.; Bates R.; Blackwell S.; Yim J.; Ronneberger O.; Bodenstein S.; Zielinski M.; Bridgland A.; Potapenko A.; Cowie A.; Tunyasuvunakool K.; Jain R.; Clancy E.; Kohli P.; Jumper J.; Hassabis D. Protein Complex Prediction with AlphaFold-Multimer. bioRxiv 2021, 2021.10.04.463034 10.1101/2021.10.04.463034.

[ref31] Mirdita M.; Schütze K.; Moriwaki Y.; Heo L.; Ovchinnikov S.; Steinegger M. ColabFold: Making Protein Folding Accessible to All. Nat. Methods 2022, 19, 679–682. 10.1038/s41592-022-01488-1. 35637307 PMC9184281

[ref32] Steinegger M.; Söding J. MMseqs. 2 Enables Sensitive Protein Sequence Searching for the Analysis of Massive Data Sets. Nat. Biotechnol. 2017, 35, 1026–1028. 10.1038/nbt.3988. 29035372

[ref33] Phan J.; Zdanov A.; Evdokimov A. G.; Tropea J. E.; Peters H. K. 3rd; Kapust R. B.; Li M.; Wlodawer A.; Waugh D. S. Structural Basis for the Substrate Specificity of Tobacco Etch Virus Protease. J. Biol. Chem. 2002, 277, 50564–50572. 10.1074/jbc.M207224200. 12377789

[ref34] Krissinel E.; Henrick K. Secondary-Structure Matching (SSM), a New Tool for Fast Protein Structure Alignment in Three Dimensions. Acta Crystallogr., Sect. D: Biol. Crystallogr. 2004, 60, 2256–2268. 10.1107/S0907444904026460. 15572779

[ref35] Krissinel E.; Henrick K. Multiple Alignment of Protein Structures in Three Dimensions. Lecture Notes in Computer Science. 2005, 67–78. 10.1007/11560500_7.

[ref36] Sun P.; Austin B. P.; Tözsér J.; Waugh D. S. Structural Determinants of Tobacco Vein Mottling Virus Protease Substrate Specificity. Protein Sci. 2010, 19, 2240–2251. 10.1002/pro.506. 20862670 PMC3005794

[ref37] Batra J.; Szabó A.; Caulfield T. R.; Soares A. S.; Sahin-Tóth M.; Radisky E. S. Long-Range Electrostatic Complementarity Governs Substrate Recognition by Human Chymotrypsin C, a Key Regulator of Digestive Enzyme Activation. J. Biol. Chem. 2013, 288, 9848–9859. 10.1074/jbc.M113.457382. 23430245 PMC3617285

[ref38] Rawlings N. D.; Barrett A. J.; Thomas P. D.; Huang X.; Bateman A.; Finn R. D. The MEROPS Database of Proteolytic Enzymes, Their Substrates and Inhibitors in 2017 and a Comparison with Peptidases in the PANTHER Database. Nucleic Acids Res. 2018, 46, D624–D632. 10.1093/nar/gkx1134. 29145643 PMC5753285

[ref39] Riley B. T.; Ilyichova O.; de Veer S. J.; Swedberg J. E.; Wilson E.; Hoke D. E.; Harris J. M.; Buckle A. M. KLK4 Inhibition by Cyclic and Acyclic Peptides: Structural and Dynamical Insights into Standard-Mechanism Protease Inhibitors. Biochemistry 2019, 58, 2524–2533. 10.1021/acs.biochem.9b00191. 31058493

[ref40] The PyMOL Molecular Graphics System, Version 2.5 Schrödinger, LLC.

[ref41] Li M.; Srp J.; Mareš M.; Wlodawer A.; Gustchina A. Structural Studies of Complexes of Kallikrein 4 with Wild-Type and Mutated Forms of the Kunitz-Type Inhibitor BbKI. Acta Crystallogr., Sect. D: Struct. Biol. 2021, 77, 1084–1098. 10.1107/S2059798321006483. 34342281 PMC8329858

[ref42] Jurrus E.; Engel D.; Star K.; Monson K.; Brandi J.; Felberg L. E.; Brookes D. H.; Wilson L.; Chen J.; Liles K.; Chun M.; Li P.; Gohara D. W.; Dolinsky T.; Konecny R.; Koes D. R.; Nielsen J. E.; Head-Gordon T.; Geng W.; Krasny R.; Wei G.; Holst M. J.; Andrew McCammon J.; Baker N. A. Improvements to the APBS Biomolecular Solvation Software Suite. Protein Sci. 2018, 27, 112–128. 10.1002/pro.3280. 28836357 PMC5734301

[ref43] Williams C. J.; Headd J. J.; Moriarty N. W.; Prisant M. G.; Videau L. L.; Deis L. N.; Verma V.; Keedy D. A.; Hintze B. J.; Chen V. B.; Jain S.; Lewis S. M.; Arendall W. B. 3rd; Snoeyink J.; Adams P. D.; Lovell S. C.; Richardson J. S.; Richardson D. C. MolProbity: More and Better Reference Data for Improved All-Atom Structure Validation. Protein Sci. 2018, 27, 293–315. 10.1002/pro.3330. 29067766 PMC5734394

[ref44] Shaqra A. M.; Zvornicanin S. N.; Huang Q. Y. J.; Lockbaum G. J.; Knapp M.; Tandeske L.; Bakan D. T.; Flynn J.; Bolon D. N. A.; Moquin S.; Dovala D.; Kurt Yilmaz N.; Schiffer C. A. Defining the Substrate Envelope of SARS-CoV-2 Main Protease to Predict and Avoid Drug Resistance. Nat. Commun. 2022, 13, 3556 10.1038/s41467-022-31210-w. 35729165 PMC9211792

[ref45] Abraham M. J.; Murtola T.; Schulz R.; Páll S.; Smith J. C.; Hess B.; Lindahl E. GROMACS: High Performance Molecular Simulations through Multi-Level Parallelism from Laptops to Supercomputers. SoftwareX 2015, 1–2, 19–25. 10.1016/j.softx.2015.06.001.

[ref46] Gillet N.; Elstner M.; Kubař T. Coupled-Perturbed DFTB-QM/MM Metadynamics: Application to Proton-Coupled Electron Transfer. J. Chem. Phys. 2018, 149, 072328 10.1063/1.5027100. 30134697

[ref47] Hourahine B.; Aradi B.; Blum V.; Bonafé F.; Buccheri A.; Camacho C.; Cevallos C.; Deshaye M. Y.; Dumitrică T.; Dominguez A.; Ehlert S.; Elstner M.; van der Heide T.; Hermann J.; Irle S.; Kranz J. J.; Köhler C.; Kowalczyk T.; Kubař T.; Lee I. S.; Lutsker V.; Maurer R. J.; Min S. K.; Mitchell I.; Negre C.; Niehaus T. A.; Niklasson A. M. N.; Page A. J.; Pecchia A.; Penazzi G.; Persson M. P.; Řezáč J.; Sánchez C. G.; Sternberg M.; Stöhr M.; Stuckenberg F.; Tkatchenko A.; Yu V. W.-Z.; Frauenheim T. DFTB+, a Software Package for Efficient Approximate Density Functional Theory Based Atomistic Simulations. J. Chem. Phys. 2020, 152, 124101 10.1063/1.5143190. 32241125

[ref48] Bussi G.; Tribello G. A. Analyzing and Biasing Simulations with PLUMED. Methods Mol. Biol. 2019, 2022, 529–578. 31396917 10.1007/978-1-4939-9608-7_21

[ref49] Tian C.; Kasavajhala K.; Belfon K. A. A.; Raguette L.; Huang H.; Migues A. N.; Bickel J.; Wang Y.; Pincay J.; Wu Q.; Simmerling C. ff19SB: Amino-Acid-Specific Protein Backbone Parameters Trained against Quantum Mechanics Energy Surfaces in Solution. J. Chem. Theory Comput. 2020, 16, 528–552. 10.1021/acs.jctc.9b00591. 31714766 PMC13071887

[ref50] Zlobin A.; Diankin I.; Pushkarev S.; Golovin A. Probing the Suitability of Different Ca Parameters for Long Simulations of Diisopropyl Fluorophosphatase. Molecules 2021, 26, 5839 10.3390/molecules26195839. 34641383 PMC8510429

[ref51] Izadi S.; Onufriev A. V. Accuracy Limit of Rigid 3-Point Water Models. J. Chem. Phys. 2016, 145, 074501 10.1063/1.4960175. 27544113 PMC4991989

[ref52] Bussi G.; Donadio D.; Parrinello M. Canonical Sampling through Velocity Rescaling. J. Chem. Phys. 2007, 126, 014101 10.1063/1.2408420. 17212484

[ref53] Bernetti M.; Bussi G. Pressure Control Using Stochastic Cell Rescaling. J. Chem. Phys. 2020, 153, 114107 10.1063/5.0020514. 32962386

[ref54] Lindorff-Larsen K.; Piana S.; Palmo K.; Maragakis P.; Klepeis J. L.; Dror R. O.; Shaw D. E. Improved Side-Chain Torsion Potentials for the Amber ff99SB Protein Force Field. Proteins 2010, 78, 1950–1958. 10.1002/prot.22711. 20408171 PMC2970904

[ref55] Gaus M.; Cui Q.; Elstner M. DFTB3: Extension of the Self-Consistent-Charge Density-Functional Tight-Binding Method (SCC-DFTB). J. Chem. Theory Comput. 2011, 7, 931–948. 10.1021/ct100684s. PMC3509502 23204947

[ref56] Gaus M.; Goez A.; Elstner M. Parametrization and Benchmark of DFTB3 for Organic Molecules. J. Chem. Theory Comput. 2013, 9, 338–354. 10.1021/ct300849w. 26589037

[ref57] Terekhov S. S.; Mokrushina Y. A.; Nazarov A. S.; Zlobin A.; Zalevsky A.; Bourenkov G.; Golovin A.; Belogurov A. Jr.; Osterman I. A.; Kulikova A. A.; Mitkevich V. A.; Lou H. J.; Turk B. E.; Wilmanns M.; Smirnov I. V.; Altman S.; Gabibov A. G. A Kinase Bioscavenger Provides Antibiotic Resistance by Extremely Tight Substrate Binding. Sci. Adv. 2020, 6, eaaz9861 10.1126/sciadv.aaz9861. 32637600 PMC7314540

[ref58] Zlobin A.; Mokrushina Y.; Terekhov S.; Zalevsky A.; Bobik T.; Stepanova A.; Aliseychik M.; Kartseva O.; Panteleev S.; Golovin A.; Belogurov A. Jr.; Gabibov A.; Smirnov I. QM/MM Description of Newly Selected Catalytic Bioscavengers Against Organophosphorus Compounds Revealed Reactivation Stimulus Mediated by Histidine Residue in the Acyl-Binding Loop. Front. Pharmacol. 2018, 9, 834 10.3389/fphar.2018.00834. 30123127 PMC6085465

[ref59] Wang X.; Li R.; Cui W.; Li Q.; Yao J. QM/MM Free Energy Simulations of an Efficient Gluten Hydrolase (Kuma030) Implicate for a Reactant-State Based Protein-Design Strategy for General Acid/Base Catalysis. Sci. Rep. 2018, 8, 7042 10.1038/s41598-018-25471-z. 29728674 PMC5935664

[ref60] Roston D.; Demapan D.; Cui Q. Extensive Free-Energy Simulations Identify Water as the Base in Nucleotide Addition by DNA Polymerase. Proc. Natl. Acad. Sci. U.S.A. 2019, 116, 25048–25056. 10.1073/pnas.1914613116. 31757846 PMC6911213

[ref61] Zlobin A. S.; Zalevsky A. O.; Mokrushina Y. A.; Kartseva O. V.; Golovin A. V.; Smirnov I. V. The Preferable Binding Pose of Canonical Butyrylcholinesterase Substrates Is Unproductive for Echothiophate. Acta Naturae 2018, 10, 121–124. 10.32607/20758251-2018-10-4-121-124. 30713771 PMC6351040

[ref62] Caldeweyher E.; Ehlert S.; Hansen A.; Neugebauer H.; Spicher S.; Bannwarth C.; Grimme S. A Generally Applicable Atomic-Charge Dependent London Dispersion Correction. J. Chem. Phys. 2019, 150, 154122 10.1063/1.5090222. 31005066

[ref63] Barducci A.; Bussi G.; Parrinello M. Well-Tempered Metadynamics: A Smoothly Converging and Tunable Free-Energy Method. Phys. Rev. Lett. 2008, 100, 020603 10.1103/physrevlett.100.020603. 18232845

[ref64] Tiwary P.; Parrinello M. A Time-Independent Free Energy Estimator for Metadynamics. J. Phys. Chem. B 2015, 119, 736–742. 10.1021/jp504920s. 25046020

[ref65] Voevodin V. V.; Antonov A. S.; Nikitenko D. A.; et al. Supercomputer Lomonosov-2: Large Scale, Deep Monitoring and Fine Analytics for the User Community. Supercomput. Front. Innovations 2019, 6, 4–11. 10.14529/jsfi190201.

[ref66] Sun Q.; Levy R. M.; Kirby K. A.; Wang Z.; Sarafianos S. G.; Deng N. Molecular Dynamics Free Energy Simulations Reveal the Mechanism for the Antiviral Resistance of the M66I HIV-1 Capsid Mutation. Viruses 2021, 13, 920 10.3390/v13050920. 34063519 PMC8156065

[ref67] Horx P.; Geyer A. Comparing the Hinge-Type Mobility of Natural and Designed Intermolecular Bi-Disulfide Domains. Front. Chem. 2020, 8, 25 10.3389/fchem.2020.00025. 32047741 PMC6997481

[ref68] Marinova V.; Wood G. P. F.; Marziano I.; Salvalaglio M. Dynamics and Thermodynamics of Ibuprofen Conformational Isomerism at the Crystal/Solution Interface. J. Chem. Theory Comput. 2018, 14, 6484–6494. 10.1021/acs.jctc.8b00702. 30359527

[ref69] Khan R. T.; Musil M.; Stourac J.; Damborsky J.; Bednar D. Fully Automated Ancestral Sequence Reconstruction Using FireProt. Curr Protoc 2021, 1, e30 10.1002/cpz1.30. 33524240

[ref70] Letunic I.; Bork P. Interactive Tree Of Life (iTOL) v5: An Online Tool for Phylogenetic Tree Display and Annotation. Nucleic Acids Res. 2021, 49, W293–W296. 10.1093/nar/gkab301. 33885785 PMC8265157

[ref71] Ciemny M.; Kurcinski M.; Kamel K.; Kolinski A.; Alam N.; Schueler-Furman O.; Kmiecik S. Protein-Peptide Docking: Opportunities and Challenges. Drug Discovery Today 2018, 23, 1530–1537. 10.1016/j.drudis.2018.05.006. 29733895

[ref72] Tsaban T.; Varga J. K.; Avraham O.; Ben-Aharon Z.; Khramushin A.; Schueler-Furman O. Harnessing Protein Folding Neural Networks for Peptide-Protein Docking. Nat. Commun. 2022, 13, 176 10.1038/s41467-021-27838-9. 35013344 PMC8748686

[ref73] Saldaño T.; Escobedo N.; Marchetti J.; Zea D. J.; Mac Donagh J.; Velez Rueda A. J.; Gonik E.; García Melani A.; Novomisky Nechcoff J.; Salas M. N.; Peters T.; Demitroff N.; Fernandez Alberti S.; Palopoli N.; Fornasari M. S.; Parisi G. Impact of Protein Conformational Diversity on AlphaFold Predictions. Bioinformatics 2022, 38, 2742–2748. 10.1093/bioinformatics/btac202. 35561203

[ref74] Buel G. R.; Walters K. J. Can AlphaFold2 Predict the Impact of Missense Mutations on Structure?. Nat. Struct. Mol. Biol. 2022, 29, 1–2. 10.1038/s41594-021-00714-2. 35046575 PMC11218004

[ref75] Jumper J.; Evans R.; Pritzel A.; Green T.; Figurnov M.; Ronneberger O.; Tunyasuvunakool K.; Bates R.; Žídek A.; Potapenko A.; Bridgland A.; Meyer C.; Kohl S. A. A.; Ballard A. J.; Cowie A.; Romera-Paredes B.; Nikolov S.; Jain R.; Adler J.; Back T.; Petersen S.; Reiman D.; Clancy E.; Zielinski M.; Steinegger M.; Pacholska M.; Berghammer T.; Bodenstein S.; Silver D.; Vinyals O.; Senior A. W.; Kavukcuoglu K.; Kohli P.; Hassabis D. Highly Accurate Protein Structure Prediction with AlphaFold. Nature 2021, 596, 583–589. 10.1038/s41586-021-03819-2. 34265844 PMC8371605

[ref76] Vanhee P.; Stricher F.; Baeten L.; Verschueren E.; Lenaerts T.; Serrano L.; Rousseau F.; Schymkowitz J. Protein-Peptide Interactions Adopt the Same Structural Motifs as Monomeric Protein Folds. Structure 2009, 17, 1128–1136. 10.1016/j.str.2009.06.013. 19679090

[ref77] Kawaguchi M.; Inoue K.; Iuchi I.; Nishida M.; Yasumasu S. Molecular Co-Evolution of a Protease and Its Substrate Elucidated by Analysis of the Activity of Predicted Ancestral Hatching Enzyme. BMC Evol. Biol. 2013, 13, 231 10.1186/1471-2148-13-231. 24161109 PMC3819744

[ref78] Kolli M.; Ozen A.; Kurt-Yilmaz N.; Schiffer C. A. HIV-1 Protease-Substrate Coevolution in Nelfinavir Resistance. J. Virol. 2014, 88, 7145–7154. 10.1128/JVI.00266-14. 24719428 PMC4054462

[ref79] Jendroszek A.; Madsen J. B.; Chana-Muñoz A.; Dupont D. M.; Christensen A.; Panitz F.; Füchtbauer E.-M.; Lovell S. C.; Jensen J. K. Biochemical and Structural Analyses Suggest That Plasminogen Activators Coevolved with Their Cognate Protein Substrates and Inhibitors. J. Biol. Chem. 2019, 294, 3794–3805. 10.1074/jbc.RA118.005419. 30651349 PMC6416416

[ref80] Lee J.; Worrall L. J.; Vuckovic M.; Rosell F. I.; Gentile F.; Ton A.-T.; Caveney N. A.; Ban F.; Cherkasov A.; Paetzel M.; Strynadka N. C. J. Crystallographic Structure of Wild-Type SARS-CoV-2 Main Protease Acyl-Enzyme Intermediate with Physiological C-Terminal Autoprocessing Site. Nat. Commun. 2020, 11, 5877 10.1038/s41467-020-19662-4. 33208735 PMC7674412

[ref81] Fontana A.; de Laureto P. P.; De Filippis V. Molecular Aspects of Proteolysis of Globular Proteins. In Studies in Organic Chemistry; Studies in Organic Chemistry; Elsevier, 1993; pp 101–110.

[ref82] Dauparas J.; Anishchenko I.; Bennett N.; Bai H.; Ragotte R. J.; Milles L. F.; Wicky B. I. M.; Courbet A.; de Haas R. J.; Bethel N.; Leung P. J. Y.; Huddy T. F.; Pellock S.; Tischer D.; Chan F.; Koepnick B.; Nguyen H.; Kang A.; Sankaran B.; Bera A. K.; King N. P.; Baker D. Robust Deep Learning-Based Protein Sequence Design Using ProteinMPNN. Science 2022, 378, eadd2187 10.1126/science.add2187. PMC9997061 36108050

[ref83] Leman J. K.; Weitzner B. D.; Lewis S. M.; Adolf-Bryfogle J.; Alam N.; Alford R. F.; Aprahamian M.; Baker D.; Barlow K. A.; Barth P.; Basanta B.; Bender B. J.; Blacklock K.; Bonet J.; Boyken S. E.; Bradley P.; Bystroff C.; Conway P.; Cooper S.; Correia B. E.; Coventry B.; Das R.; De Jong R. M.; DiMaio F.; Dsilva L.; Dunbrack R.; Ford A. S.; Frenz B.; Fu D. Y.; Geniesse C.; Goldschmidt L.; Gowthaman R.; Gray J. J.; Gront D.; Guffy S.; Horowitz S.; Huang P.-S.; Huber T.; Jacobs T. M.; Jeliazkov J. R.; Johnson D. K.; Kappel K.; Karanicolas J.; Khakzad H.; Khar K. R.; Khare S. D.; Khatib F.; Khramushin A.; King I. C.; Kleffner R.; Koepnick B.; Kortemme T.; Kuenze G.; Kuhlman B.; Kuroda D.; Labonte J. W.; Lai J. K.; Lapidoth G.; Leaver-Fay A.; Lindert S.; Linsky T.; London N.; Lubin J. H.; Lyskov S.; Maguire J.; Malmström L.; Marcos E.; Marcu O.; Marze N. A.; Meiler J.; Moretti R.; Mulligan V. K.; Nerli S.; Norn C.; Ó’Conchúir S.; Ollikainen N.; Ovchinnikov S.; Pacella M. S.; Pan X.; Park H.; Pavlovicz R. E.; Pethe M.; Pierce B. G.; Pilla K. B.; Raveh B.; Renfrew P. D.; Burman S. S. R.; Rubenstein A.; Sauer M. F.; Scheck A.; Schief W.; Schueler-Furman O.; Sedan Y.; Sevy A. M.; Sgourakis N. G.; Shi L.; Siegel J. B.; Silva D.-A.; Smith S.; Song Y.; Stein A.; Szegedy M.; Teets F. D.; Thyme S. B.; Wang R. Y.-R.; Watkins A.; Zimmerman L.; Bonneau R. Macromolecular Modeling and Design in Rosetta: Recent Methods and Frameworks. Nat. Methods 2020, 17, 665–680. 10.1038/s41592-020-0848-2. 32483333 PMC7603796

[ref84] Del Alamo D.; Sala D.; Mchaourab H. S.; Meiler J. Sampling Alternative Conformational States of Transporters and Receptors with AlphaFold2. eLife 2022, 11, e75751 10.7554/eLife.75751. 35238773 PMC9023059

[ref85] Toul M.; Nikitin D.; Marek M.; Damborsky J.; Prokop Z. Extended Mechanism of the Plasminogen Activator Staphylokinase Revealed by Global Kinetic Analysis: 1000-Fold Higher Catalytic Activity than that of Clinically Used Alteplase. ACS Catal. 2022, 12, 3807–3814. 10.1021/acscatal.1c05042.

[ref86] Pelc L. A.; Koester S. K.; Kukla C. R.; Chen Z.; Di Cera E. The Active Site Region Plays a Critical Role in Na Binding to Thrombin. J. Biol. Chem. 2022, 298, 101458 10.1016/j.jbc.2021.101458. 34861239 PMC8695361

[ref87] Gianni S.; Ivarsson Y.; Bah A.; Bush-Pelc L. A.; Di Cera E. Mechanism of Na(+) Binding to Thrombin Resolved by Ultra-Rapid Kinetics. Biophys. Chem. 2007, 131, 111–114. 10.1016/j.bpc.2007.09.009. 17935858 PMC2111677

[ref88] Kneller D. W.; Phillips G.; Weiss K. L.; Pant S.; Zhang Q.; O’Neill H. M.; Coates L.; Kovalevsky A. Unusual Zwitterionic Catalytic Site of SARS-CoV-2 Main Protease Revealed by Neutron Crystallography. J. Biol. Chem. 2020, 295, 17365–17373. 10.1074/jbc.AC120.016154. 33060199 PMC7832724

