# Supplementary material for: Between Protein Fold and Nucleophile Identity: Multiscale Modeling of the TEV Protease Enzyme–Substrate Complex
Source: ACS Omega. 2022 Oct 27;7(44):40279–92. doi: 10.1021/acsomega.2c05201 (PMC9647873; doi:10.1021/acsomega.2c05201)
Supplement: Supplementary file 1 — ao2c05201_si_001.pdf [file ao2c05201_si_001.pdf]

SUPPORTING INFORMATION

Between protein fold and nucleophile identity: multiscale modeling of TEV protease enzyme-substrate complex

Alexander Zlobin[1,2,*], Andrey Golovin[1,2,3*]

1. Belozersky Institute of Physico-Chemical Biology, Lomonosov Moscow State University, 119991 Moscow, Russia
2. Shemyakin and Ovchinnikov Institute of Bioorganic Chemistry, Russian Academy of Sciences, 117997 Moscow, Russia
3. Sirius University of Science and Technology, 354340 Sochi, Russia

* Correspondence alexander.zlobin@fbb.msu.ru, golovin@fbb.msu.ru

In this document additional figures are available:

- S1. Fold and active site differences between PA proteases and papain;
- S2. QM region composition;
- S3, S4. Convergence and reproducibility in QM/MM metadynamics runs;
- S5. Comparison of MetaD results when initiating from six independent 10 ns equilibration runs and from 6 frames taken after the 1 μs of a 2 μs run.
- S5. Comparison of QM/MM and MM reconstructions of Ser168 torsion free energy profile;
- S6. Free energy profiles of Ser CA-CB rotation from MM runs;
- S7. Convergence of MM metadynamics runs tracked by free energy difference between Ser states
- S8. Comparison of free energy profiles of Ser168 rotation obtained from QM/MM and pure MM calculations
- S9. Hydration in the active site of serine PA-clan proteases in a peptide-bound form
- S10. Phylogenetic tree of viral cysteine PA clan proteases;
- S11. AlphaFold2 models from trimmed alignments;
- S12. Showcase of different acid identity and architecture across distant viral PA clan cysteine proteases.

Supplemental Data including example states from QM/MM metadynamics runs, AlphaFold2-multimer-produced models and inputted multiple sequence alignments, input files for Plumed and FireProt-ASR output is deposited on Zenodo and can be accessed by DOI: 10.5281/zenodo.7093045.

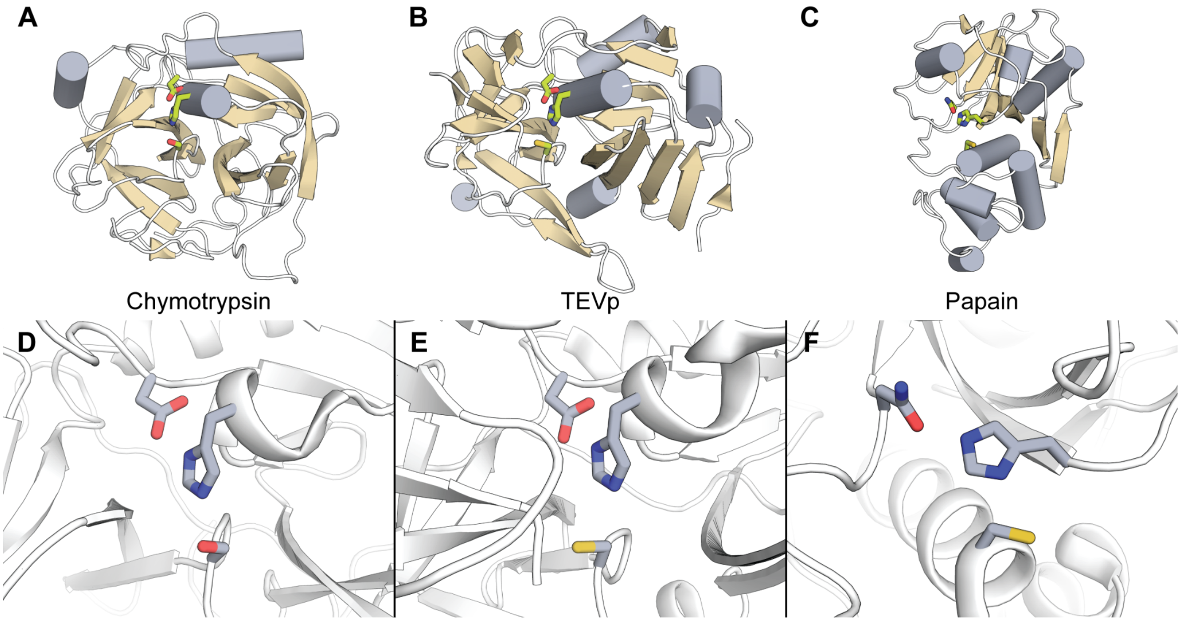

Figure S1. Similarities and differences in fold and triad architectures between chymotrypsin, TEVp and papain. A. Overall fold of chymotrypsin showing two archetypal beta-barrels. **B.** Overall fold of TEVp showing two distorted archetypal beta-barrels of chymotrypsin-like fold. **C.** Overall fold of papain with nothing in common to chymotrypsin-like folds. **D-E.** Catalytic triad relative orientations in chymotrypsin (D), TEVp (E), papain (F). For a way to describe and quantify such relative orientations please refer to ref. 24 of main text.

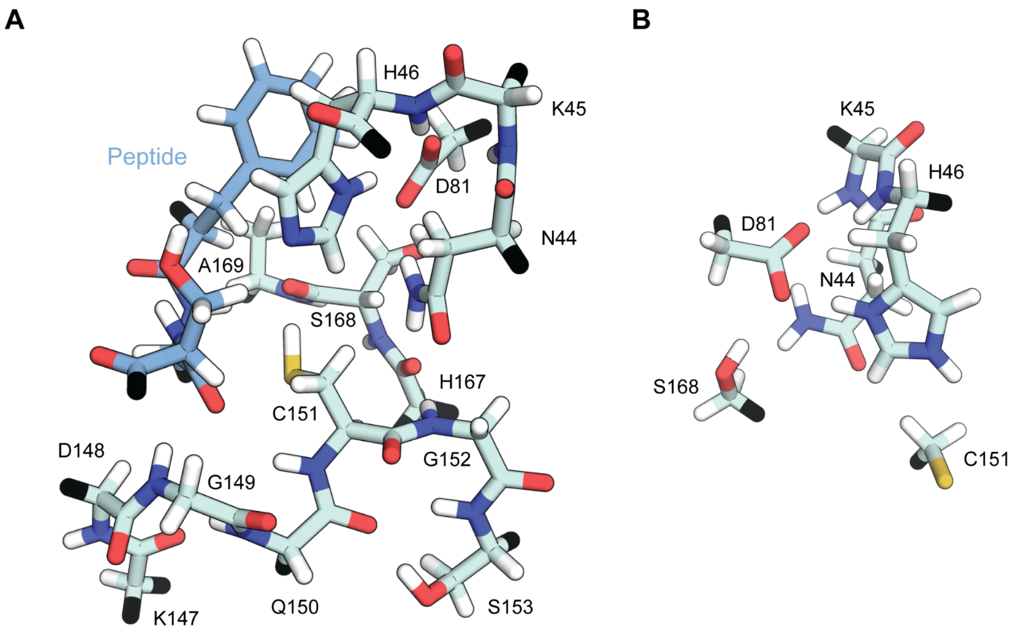

Figure S2. QM region compositions used in this work. A. For peptide-bound state calculations. **B**. For free enzyme calculations. Link atoms are shown in black.

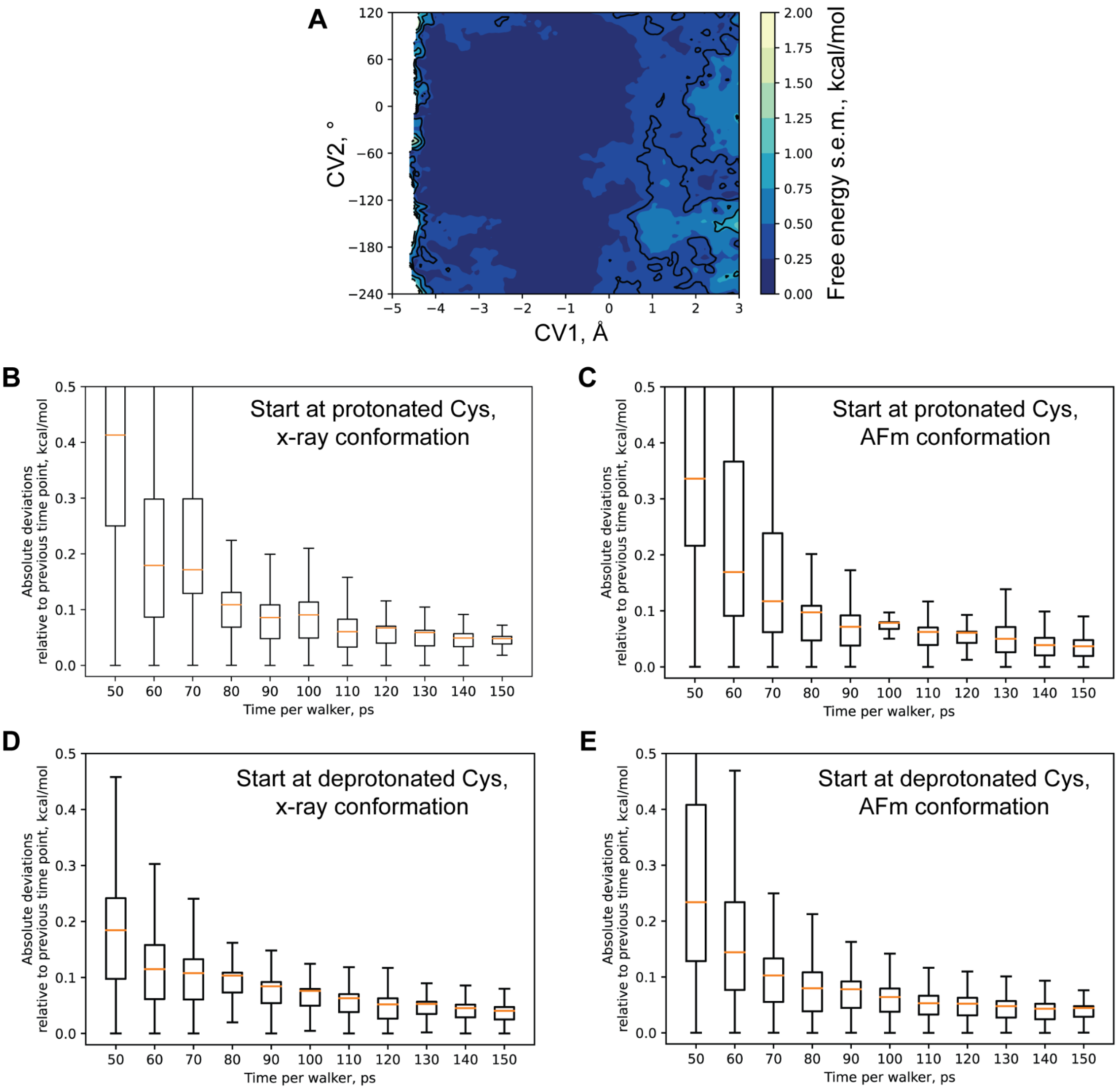

Figure S3. Convergence and reproducibility in peptide-bound QM/MM metadynamics calculations. A. Reproducibility shown as free energy profile standard errors of mean from 4 profiles obtained by starting runs from different initial Cys protonation and Ser168 geometry. **B-E.** Convergence for individual runs as absolute deviations between corresponding grid cells. Free energy profiles were constructed by cumulative addition of data from 20ps to a particular time, and deviations were calculated relative to the previous time point.

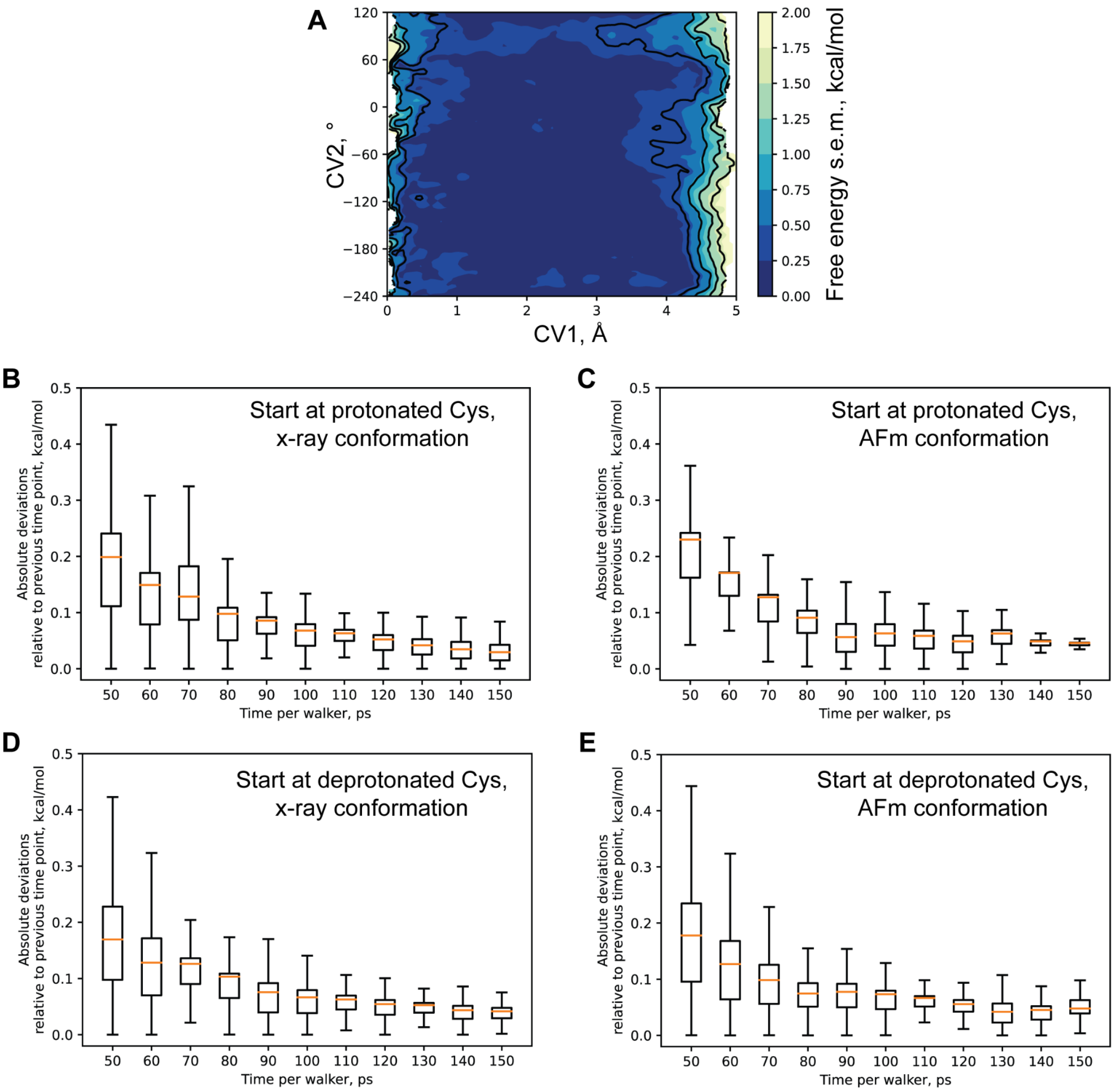

Figure S4. Convergence and reproducibility in free enzyme QM/MM metadynamics calculations. A. Reproducibility shown as free energy profile standard errors of mean from 4 profiles obtained by starting runs from different initial Cys protonation and Ser168 geometry. **B-E.** Convergence for individual runs as absolute deviations between corresponding grid cells. Free energy profiles were constructed by cumulative addition of data from 20ps to a particular time, and deviations were calculated relative to the previous time point.

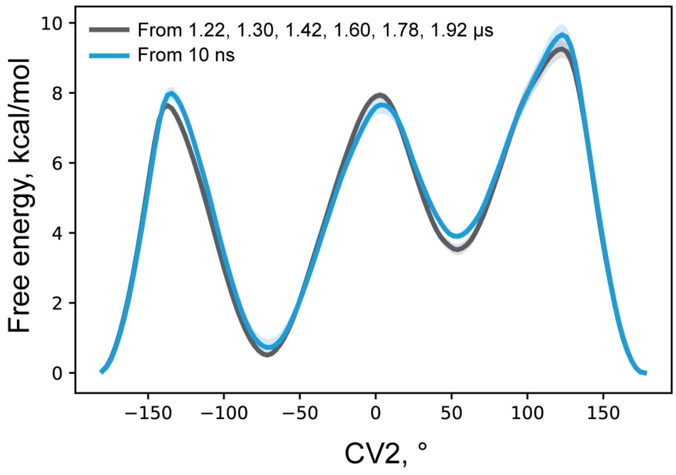

Figure S5. Comparison of MetaD results when initiating from six independent 10 ns equilibration runs and from 6 frames taken after the 1 μs of a 2 μs run.

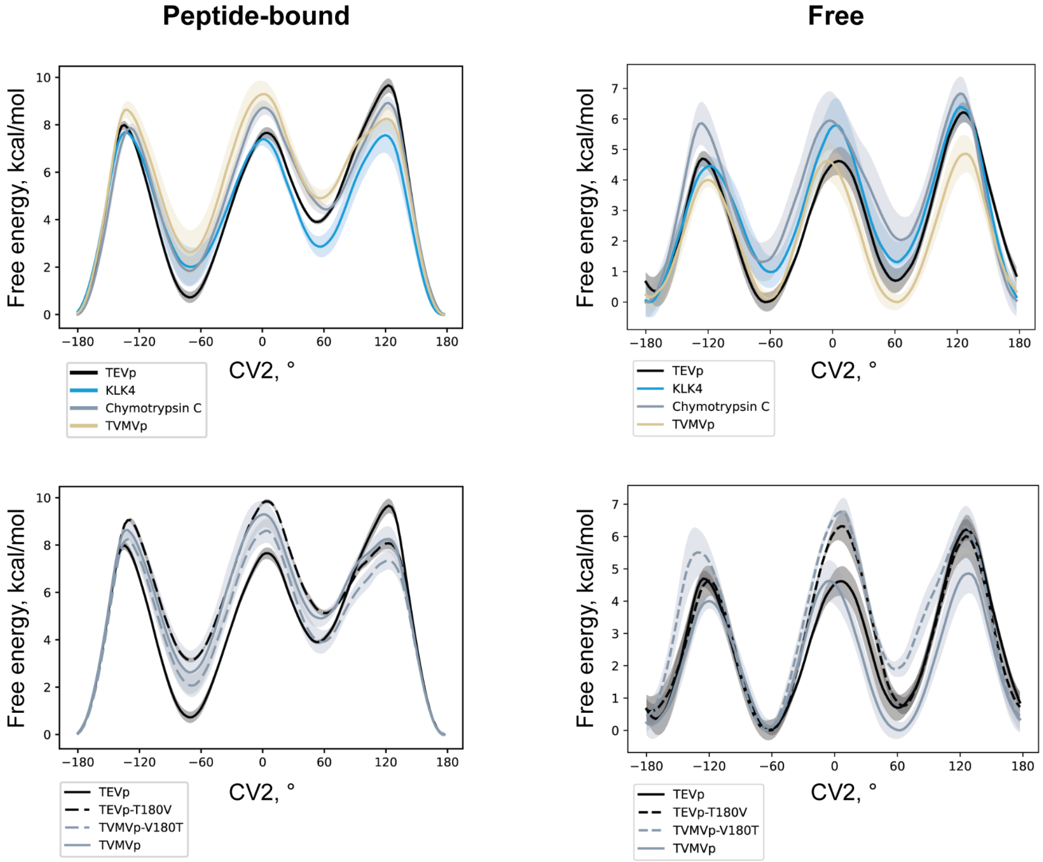

Figure S6. Free energy profiles of Ser CA-CB rotation from MM runs.

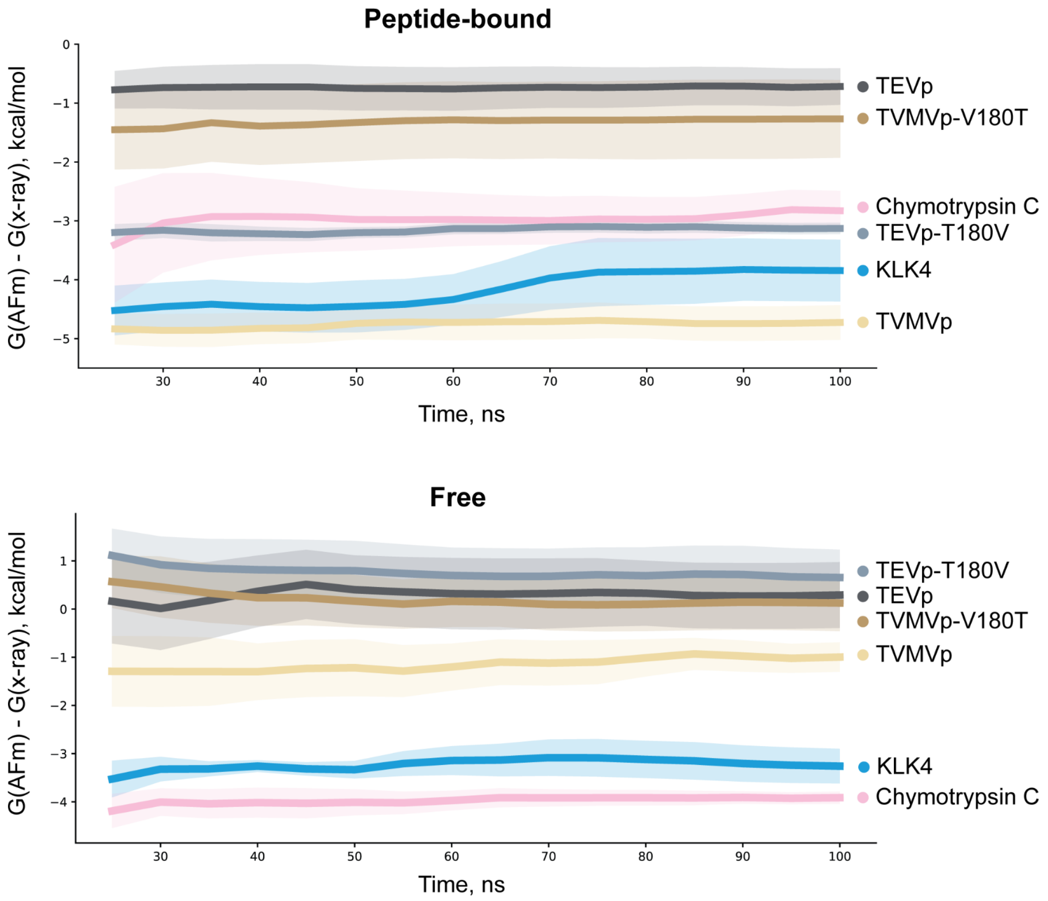

Figure S7. Convergence of MM metadynamics runs tracked by free energy difference between Ser states.

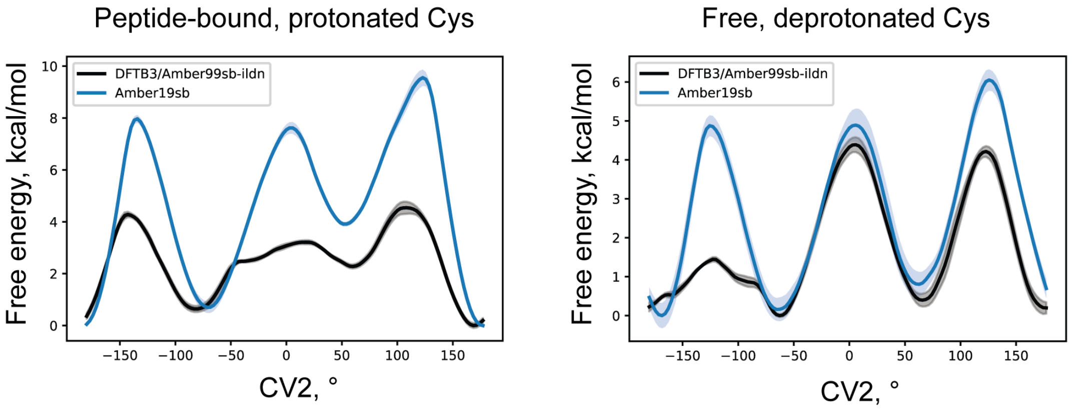

Figure S8. Comparison of free energy profiles of Ser168 rotation obtained from QM/MM and pure MM calculations. Solid line is a mean from 3 replicates, shaded area represents standard error of mean.

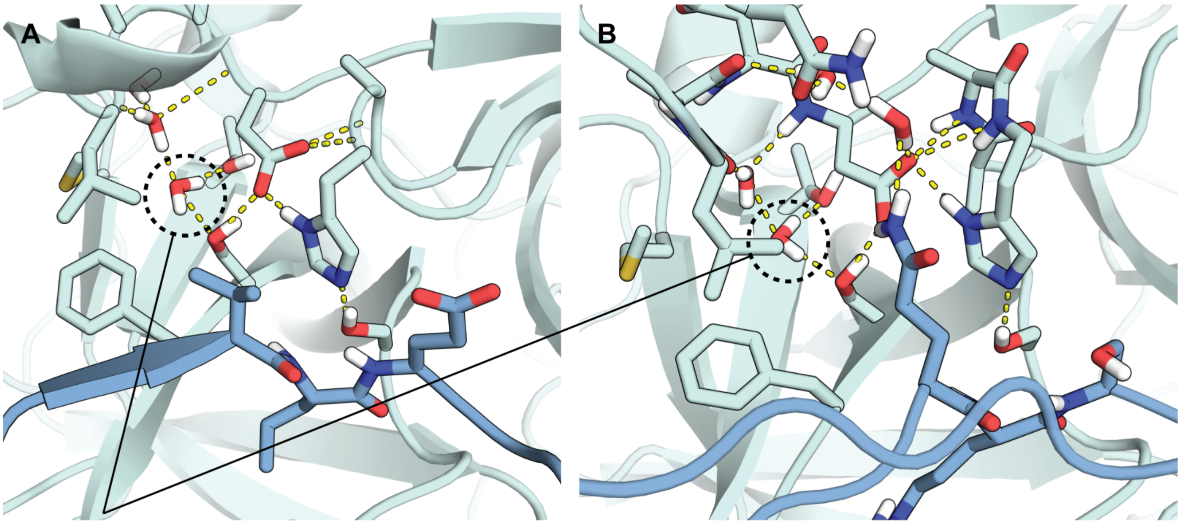

Figure S9. Hydration in the active site of serine PA-clan proteases in a peptide-bound form. **A**. Chymotrypsin C. **B**. Kallikrein 4.

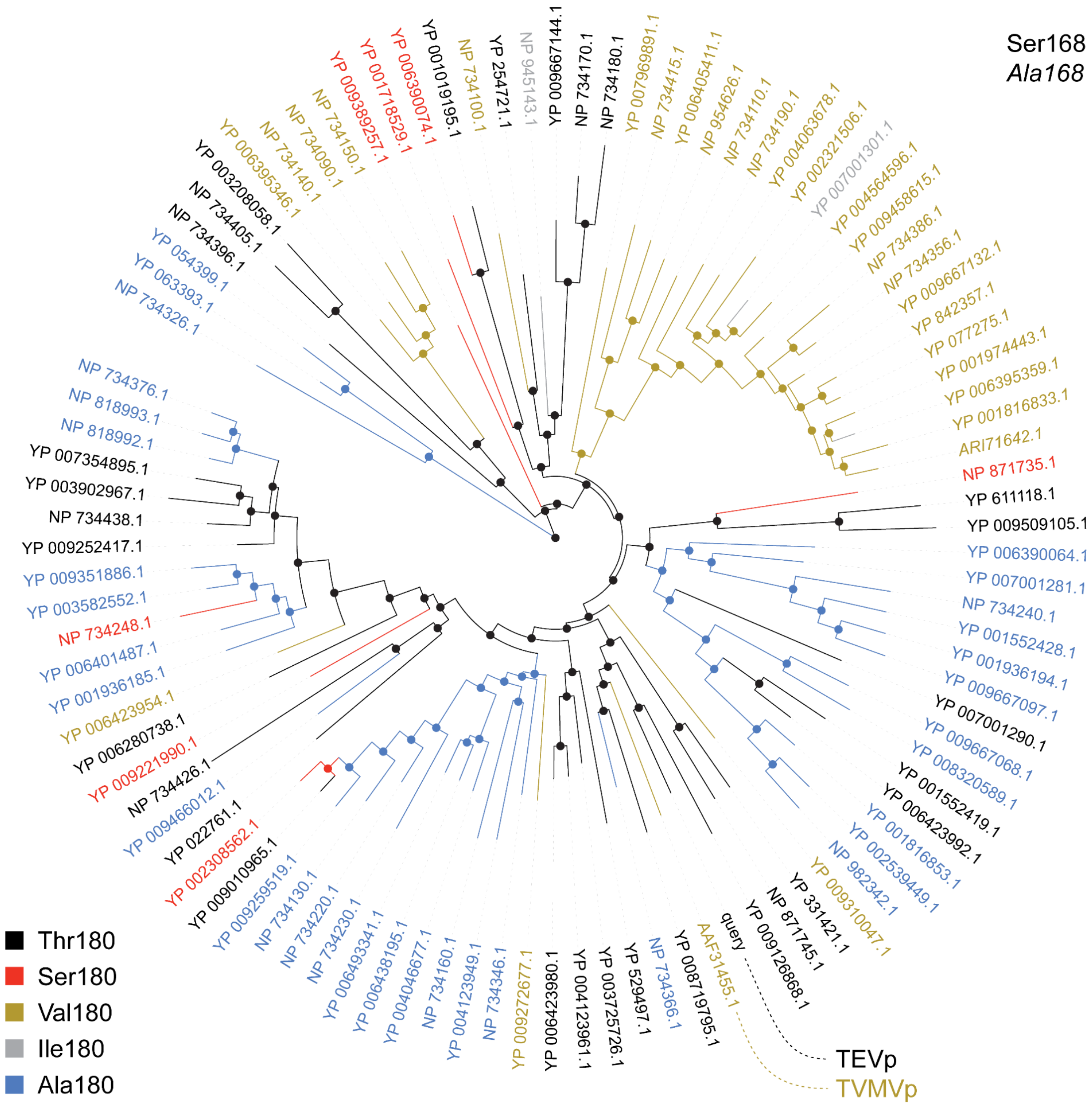

Figure S10. Phylogenetic analysis of viral PA-clan Cys proteases. Circles represent reconstructed ancestors.

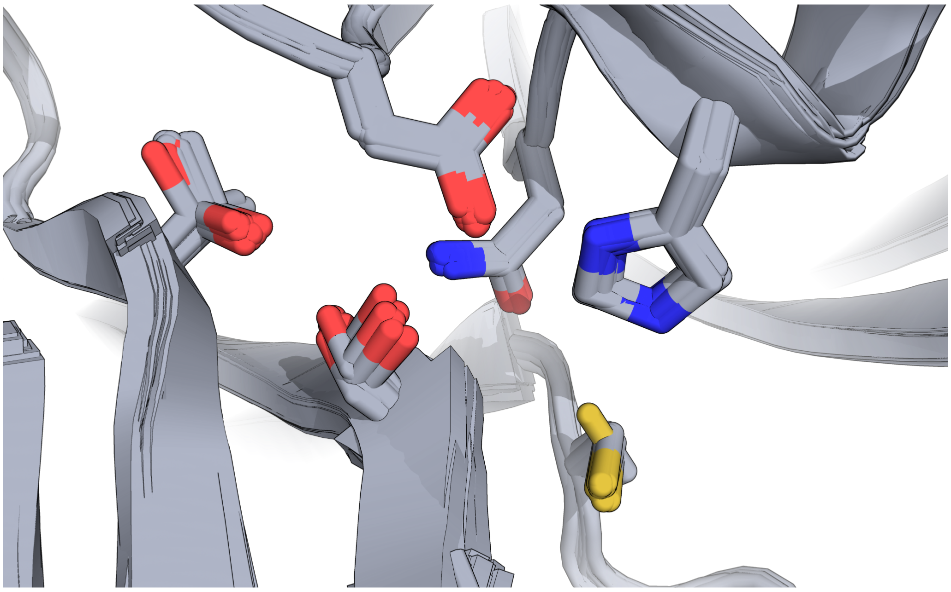

Figure S11. Overlay of 30 AlphaFold models constructed from trimmed alignments.

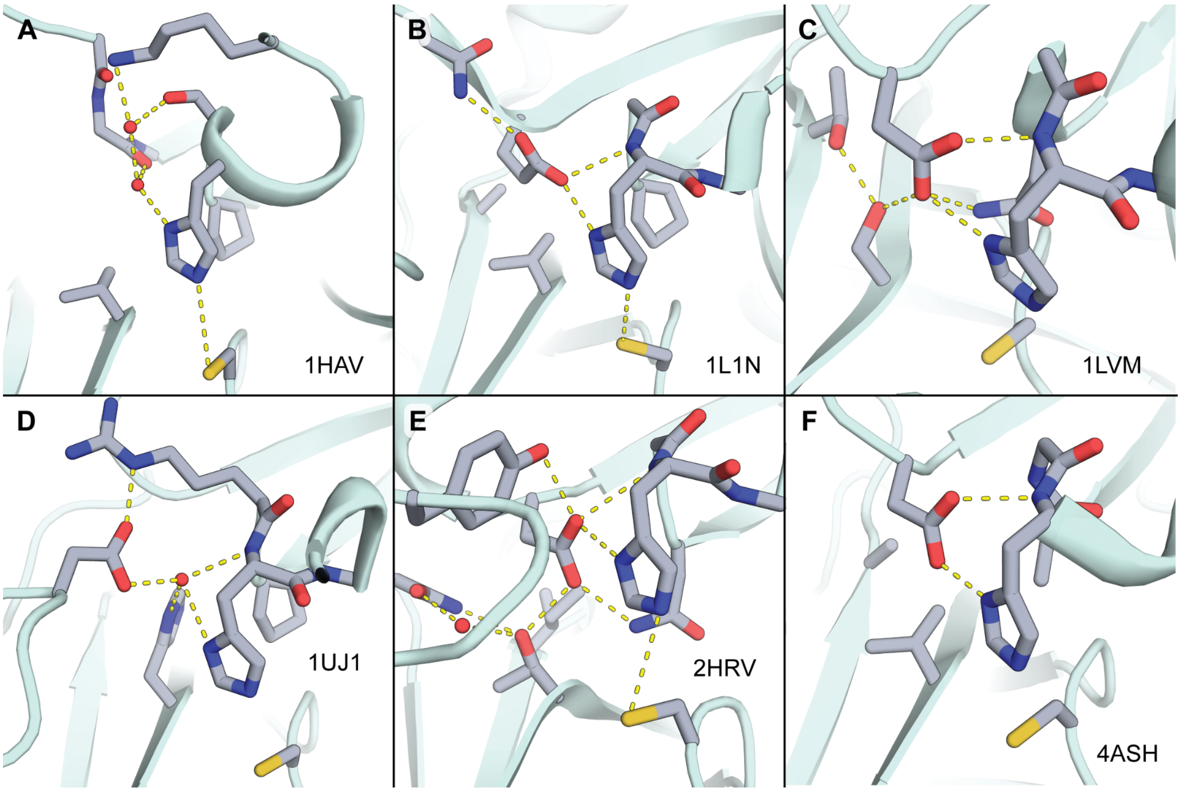

Figure S12. Examples of dramatic variation in catalytic triad acid identity, conformation and surroundings in different PA-clan Cys proteases. A. Hepatitis A virus 3C protease, PDB ID 1HAV. **B.** Poliovirus 3C protease, PDB ID 1L1N. **C.** Tobacco Etch Virus protease, PDB ID 1LVM. **D.** 3CLpro from SARS coronavirus, PDB ID 1UJ1. **E.** Rhinovirus 2 2A cysteine protease, PDB ID 2HRV. **F.** NS6 protease from murine norovirus 1, PDB ID 4ASH.
